# The ARPKD Protein DZIP1L Regulates Ciliary Protein Entry by Modulating the Architecture and Function of Ciliary Transition Fibers

**DOI:** 10.1002/advs.202308820

**Published:** 2024-04-17

**Authors:** Huicheng Chen, Zhimao Wu, Ziwei Yan, Chuan Chen, Yingying Zhang, Qiaoling Wang, Yuqing Gao, Kun Ling, Jinghua Hu, Qing Wei

**Affiliations:** ^1^ CAS Key Laboratory of Insect Developmental and Evolutionary Biology CAS Center for Excellence in Molecular Plant Sciences Chinese Academy of Sciences Shanghai 200032 China; ^2^ University of Chinese Academy of Sciences Beijing 100039 China; ^3^ Center for Energy Metabolism and Reproduction Institute of Biomedicine and Biotechnology Shenzhen Institutes of Advanced Technology Chinese Academy of Sciences (CAS) Shenzhen 518055 China; ^4^ Department of Biochemistry and Molecular Biology Mayo Clinic Rochester MN 55905 USA; ^5^ Institute of Medicine and Pharmaceutical Sciences Zhengzhou University Zhengzhou 430000 China; ^6^ School of Synthetic Biology Shanxi Key Laboratory of Nucleic Acid Biopesticides Shanxi University Taiyuan 030006 China

**Keywords:** ANKRD26, autosomal recessive polycystic kidney disease (ARPKD), DZIP1L, transition fibers (TFs)

## Abstract

Serving as the cell's sensory antennae, primary cilia are linked to numerous human genetic diseases when they malfunction. DZIP1L, identified as one of the genetic causes of human autosomal recessive polycystic kidney disease (ARPKD), is an evolutionarily conserved ciliary basal body protein. Although it has been reported that DZIP1L is involved in the ciliary entry of PKD proteins, the underlying mechanism remains elusive. Here, an uncharacterized role of DZIP1L is reported in modulating the architecture and function of transition fibers (TFs), striking ciliary base structures essential for selective cilia gating. Using *C. elegans* as a model, C01G5.7 (hereafter termed DZIP‐1) is identified as the sole homolog of DZIP1L, which specifically localizes to TFs. While DZIP‐1 or ANKR‐26 (the ortholog of ANKRD26) deficiency shows subtle impact on TFs, co‐depletion of DZIP‐1 and ANKR‐26 disrupts TF assembly and cilia gating for soluble and membrane proteins, including the ortholog of ADPKD protein polycystin‐2. Notably, the synergistic role for DZIP1L and ANKRD26 in the formation and function of TFs is highly conserved in mammalian cilia. Hence, the findings illuminate an evolutionarily conserved role of DZIP1L in TFs architecture and function, highlighting TFs as a vital part of the ciliary gate implicated in ciliopathies ARPKD.

## Graphical abstract

As a genetic cause of human autosomal recessive polycystic kidney disease (ARPKD), DZIP1L is implicated in the ciliary entry of PKD proteins, yet the underlying mechanism remains elusive. This study unveils an evolutionarily conserved role of DZIP1L in modulating the architecture and function of ciliary transition fibers (TFs), highlighting TFs as a vital part of the selective ciliary gate implicated in ciliopathies ARPKD.

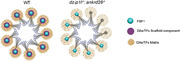

## 1 Introduction

Most eukaryotic cells have microtubule‐based sensory devices known as primary cilia that extend from their surface. Intraflagellar transport (IFT) is responsible for constructing and maintaining all cilia.^[^ ^1^, ^2^ ^]^ These structures act as signaling centers that enable cells to respond to environmental cues, regulating processes such as proliferation, differentiation, and tissue homeostasis.^[^ ^3^, ^4^, ^5^, ^6^ ^]^ So far, at least 35 human rare genetic disorders, including ADPKD and ARPKD, have been characterized as ciliopathies.^[^ ^7^, ^8^, ^9^ ^]^ These syndromic disorders collectively affect most organs in human bodies, with renal anomalies like polycystic kidneys being a common feature. Despite their significance, the mechanisms by which dysregulated ciliopathy proteins cause these manifestations remain poorly understood.

The ciliary lumen is distinct from other cellular organelles as it remains open to the cytoplasm, thus needs a hypothetical gate located at the ciliary base for selective import/export of ciliogenic proteins essential for cilia formation and function. Transition fibers (TFs), the pinwheel‐like first physical structures between the cytoplasm and the ciliary lumen, have been suggested to fulfill this critical function.^[^ ^10^, ^11^ ^]^ Currently, more than 10 proteins have been shown to localize on TFs.^[^ ^12^, ^13^, ^14^, ^15^, ^16^, ^17^, ^18^ ^]^ Among them, CEP89, SCLT1, CEP164 and ANKRD26 are likely the structural basis necessary for forming the individual fibers connecting the basal body and the ciliary membrane.^[^ ^14^ ^]^ TFs have diverse functions in cilia biology. They are crucial during the initiation of ciliogenesis as they facilitate the recruitment of ciliary vesicles, docking of the basal body, and the onset of ciliogenesis.^[^ ^14^, ^19^, ^20^ ^]^ Additionally, the amorphous space between individual fibers is believed to be a critical part of the ciliary gate in mature cilia.^[^ ^11^, ^21^, ^22^ ^]^ Recent research has shown that TFs are indispensable for the selective gating of both membrane and soluble proteins into cilia.^[^ ^15^, ^22^, ^23^, ^24^ ^]^

We have identified DYF‐19 and its mammalian ortholog FBF1 as the first functional components of TFs.^[^ ^15^, ^25^ ^]^ FBF1 deficiency impairs gating function but not fiber structure.^[^ ^15^, ^23^, ^24^ ^]^ Using DYF‐19 as a marker, we further identified additional TF functional components/regulators in *C. elegans*, including orthologs of human ciliopathy or disease proteins ANKRD26, TALPID3, and HYLS1.^[^ ^15^, ^23^, ^24^ ^]^ Of note, these newly identified TF related components play a highly conserved role in regulating cilia gating during evolution.^[^ ^15^, ^23^, ^24^, ^25^ ^]^ Despite this progress, much still remains unclear about the full composition of TFs and how TFs are established as a functional cilia gate.

The DZIP1L protein has been linked to ARPKD and reported as a protein associated with the transition zone (TZ) in ciliated organisms and implicated in ciliogenesis regulation.^[^ ^26^, ^27^ ^]^ All identified members of the DZIP1L family feature a poorly understood DZIP‐like domain at the N‐terminus. The two ARPKD mutations, p.A90V and p.Q91H^[^ ^26^ ^]^ of DZIP1L locate in the highly conserved DZIP‐like domain. Here, by using the DZIP‐like domain in a BLAST analysis, we identified *C. elegans* gene *C01G5.7* (referred to as *dzip‐1*) as the potential homolog of human DZIP1L. Unexpectedly, DZIP‐1 specifically localizes to TFs but not the TZ in both worm and human cilia. While deficiency of DZIP‐1 alone in *C. elegans* had only a mild impact on ciliogenesis and cilia gating, its combined deletion with *ankr‐26* severely impaired TF architecture and disrupted cilia gating for IFT machineries and membrane receptors, including PKD‐2, which is the homolog of the human ADPKD protein polycystin‐2. We further revealed that the genetic interaction between DZIP1L and ANKRD26 in building a functional cilia gate is highly conserved in human cilia. This sheds light on understanding the pathogenesis of DZIP1L‐associated ARPKD. Our results thus not only reveal DZIP1L as a key player in regulating the poorly understood cilia trafficking of polycystins, but also underscore the physiological significance of a functional cilia gate in the context of ciliopathies beyond PKDs.

## 2 Results

### 2.1 C. Elegans DZIP‐1 is a Genuine TF Component

**Figure 1:**
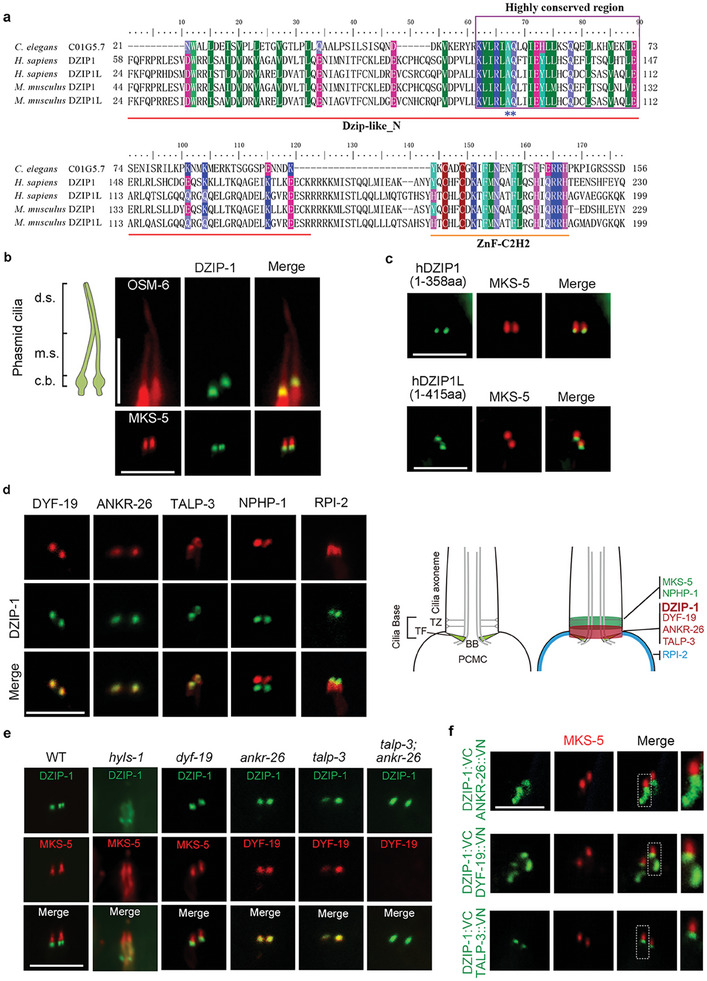
*C. elegans* DZIP‐1 is an evolutionarily conserved basal body protein. A) The sequence alignment of two evolutionarily conserved domain (DZIP‐like domain and the C2H2 zine finger domain) within *C. elegans* DZIP‐1, *H. sapiens* DZIP1/DZIP1L and *M. musculus* DZIP1/DZIP1L. The Dzip‐like_N domain (red line) is the most conserved domain in DZIP1/DZIP1L proteins. The ZnF_C2H2 domain is indicated by an orange line. And the highly conserved region is enclosed in the purple box. Asterisks are used to indicate the residues that, when mutated, can cause human ARPKD. B) DZIP‐1 localizes to the ciliary base in *C. elegans*. The schematic diagram of phasmid cilia is depicted on the left, GFP‐tagged DZIP‐1 specifically localizes to the base of cilia indicated by IFT‐B protein IFT52/OSM‐6::GFP, and localizes below the TZ marker MKS‐5. Scale bars: 5 µm. C) GFP‐tagged N‐terminal sequences of *H. sapiens* DZIP1 and DZIP1L were observed specifically localizes to the ciliary base in *C. elegans*. D) Spatial relationships between DZIP‐1 and ciliary base proteins in *C. elegans*. DZIP‐1 localizes below the TZ protein NPHP‐1, above the PCMC protein RPI‐2, and colocalizes with the TF related proteins DYF‐19, ANKR‐26, and TALP‐3. The schematics are shown in the right panel. E) HYLS‐1 is required for the ciliary base localization of DZIP‐1. F) In *vivo* interactions among DZIP‐1, ANKR‐26, TALP‐3 and DYF‐19 were observed using the bimolecular fluorescence complementation (BiFC) assay. Scale bars: 5 µm.

To search for potential worm homolog of human DZIP1L, we used the highly conserved DZIP‐like domain and the C2H2 zine finger domain in a BLAST analysis. The uncharacterized *C. elegans* gene *C01G5.7*, despite encoding a protein significantly shorter than human DZIP1L (156 a.a. vs 756 a.a.), was identified as the sole candidate gene that contains both conserved domains (**Figure** **1**a). Further analyses show that C01G5.7 protein localizes to the ciliary base in both phasmid and amphid cilia of *C. elegans* (Figure 1b, Figure S1a, Supporting Information). Notably, either the truncated N‐terminus of human DZIP1L (1‐415 aa) or DZIP1 (1‐358 aa) faithfully localizes to the ciliary base in worm cilia (Figure 1c; Figure S1b, Supporting Information). Given the close similarity in protein architecture and conserved cilia localization, we hereafter named *c01g5.7* as *dzip‐1* for ease of reference.

Similar to mammalian cilia, the ciliary base of *C. elegans* is composed of distinct compartments, including the periciliary membrane compartment (PCMC), basal body/transition fibers (TFs), and the transition zone (TZ) (Figure 1d).^[^ ^28^ ^]^ GFP‐tagged DZIP‐1 was found to completely overlap with TF component DYF‐19 (the human FBF1 ortholog), ANKR‐26, and TALP‐3 (the human TALPID‐3 ortholog), but was located below the TZ markers MKS‐5 and NPHP‐1, and above the PCMC component RPI‐2 (Figure 1d). We previously showed that a deficiency of HYLS‐1, the worm homolog of human hydrolethalus syndrome protein HYLS1,^[^ ^24^ ^]^ impairs the proper localization of all known TF components in *C. elegans*. In *hyls‐1* mutants, DZIP‐1 localization was severely disrupted, with the signal appearing within the PCMC region (Figure 1e). However, the deficiency of either TF proteins or TZ proteins did not impact the localization of DZIP‐1 (Figure 1e; Figure S1c, Supporting Information). ANKR‐26 and TALP‐3 were previously found to work together upstream of DYF‐19 to regulate TF targeting of DYF‐19 and cilia gating.^[^ ^23^ ^]^ Of note, DZIP‐1 localization was not impacted in *ankr‐26; talp‐3* double mutant cilia (Figure 1e). Taken together, these results suggest that *C. elegans* DZIP‐1 is a TF related protein.

### 2.2 In Vivo Association Between DZIP‐1 and TF Components

We then used bimolecular fluorescence complementation (BiFC) assay, an imaging approach visualize in vivo protein‐protein interaction,^[^ ^29^ ^]^ to investigate the in vivo association between DZIP‐1 and other TF‐related proteins. Our results indeed demonstrated the anticipated complementary fluorescent signals between DZIP‐1 and ANKR‐26, TALP‐3, or DYF‐19 at TFs in living animals (Figure 1f). It is noteworthy that complementary fluorescent signals between DZIP‐1 and ANKR‐26 or DYF‐19, but not with TALP‐3, could be observed in the PCMC. Similar PCMC BiFC signals were also found in our previous studies of TALP‐3 and ANKR‐26.^[^ ^23^ ^]^ It is either an artifact caused by overexpression of protein of interests, or DZIP1L associates with other TF proteins along the trafficking route to TFs. Nonetheless, the BiFC assays strongly support that DZIP‐1 is core TF component closely associated with all other TF proteins in *C. elegans* cilia.

### 2.3 Diseases Associated Mutations Affect the Basal Body Localization of DZIP‐1

The poorly characterized DZIP‐like domain contains a 28 amino acid sequence that is conserved across species and contains two mutations (p.Ala90Val and p.Gln91His) associated with ARPKD (Figure 1a). To investigate how these mutations affect DZIP1L, we engineered a worm mutant protein (DZIP‐1^AQ2VH^) with both mutations (A67V and Q68H). The results showed that DZIP‐1^AQ2VH^::GFP failed to localize to TFs, indicating that the mutations are critical for the basal body localization of DZIP‐1 (Figure S1a, Supporting Information).

### 2.4 DZIP‐1 Modulates the Architecture of TFs/Basal Body in *C. Elegans*

**Figure 2:**
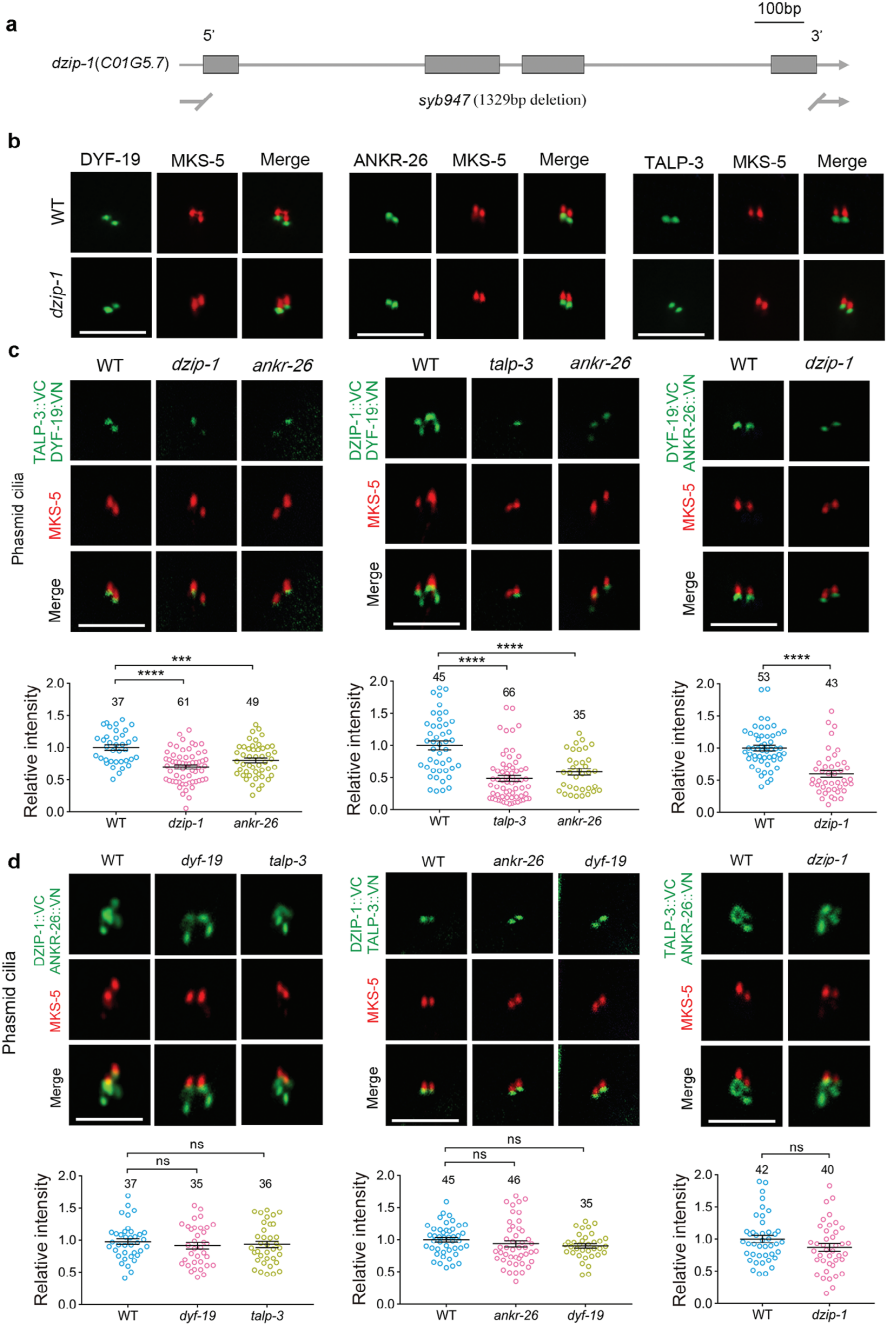
DZIP‐1 modulates the architecture of TFs/basal body in *C. elegans*. A) Schematic diagram of the genome structure of *C. elegans dzip‐1* (C01G5.7). The entire of DNA sequence (1329bp) of *dzip‐1* gene was completely knocked out by CRISPR‐Cas9 gene editing in our *dzip‐1 (syb947)* mutant. B) DZIP‐1 is dispensable for the basal body localization of other TF‐related proteins. C) BiFC signals between DYF‐19 and other TF components were observed in indicated genetic background. The fluorescence complementation signal between DYF‐19 and TALP‐3 was disrupted in *dzip‐1* or *ankr‐26* single mutants. Similarly, the fluorescence complementation signal between DYF‐19 and DZIP‐1 was disrupted in *talp‐3* or *ankr‐26* single mutant, and the fluorescence complementation signal between ANKR‐26 and DYF‐19 was also disrupted in *dzip‐1* single mutant. D) BiFC signals among DZIP‐1, ANKR‐26, and TALP‐3 were observed in indicated genetic background. In *dyf‐19* or *talp‐3* single mutants, the fluorescence complementation signal between DZIP‐1 and ANKR‐26 was comparable to that in WT. Similarly, in *ankr‐26* or *dyf‐19* single mutants, the fluorescence complementation signal between TALP‐3 and DZIP‐1 was comparable to that in WT. In *dzip‐1* single mutant, the fluorescence complementation signal between ANKR‐26 and TALP‐3 was also comparable to that in WT. Scale bars: 5 µm.

To investigate the role of DZIP‐1 in the context of TFs in *C. elegans*, we used CRISPR/Cas9‐mediated genome editing technology to knock out the full coding region of the *dzip‐1* gene (**Figure** **2**a). Examination of the localization of TF proteins in *dzip‐1 null* mutants showed that they were still targeted to the basal body, indicating that DZIP‐1 alone is dispensable for the basal body targeting of individual TF components (Figure 2b). Interestingly, we observed that the BiFC signal between DYF‐19 and other TF components was significantly reduced in *dzip‐1 null* mutants (Figure 2c; Figure S2a, Supporting Information), suggesting that deletion of DZIP‐1 alters TF architecture. Similarly, deletion of either TALP‐3 or ANKR‐26 also affects the association between DYF‐19 and the other two TF components (Figure 2c; Figure S2a, Supporting Information). However, deletion of DYF‐19 does not affect the association among DZIP‐1, ANKR‐26, and TALP‐3 (Figure 2d; Figure S2b, Supporting Information). Thus, DYF‐19 is likely incorporated into TFs after the assembly of the DZIP‐1‐ANKR‐26‐TALP‐3 subcomplex, and the loss of any protein among the DZIP‐1‐ANKR‐26‐TALP‐3 subcomplex will affect the architecture of TFs, especially the proper incorporation of DYF‐19 to the formed cilia gate.

### 2.5 DZIP‐1 Genetically Interacts with ANKR‐26 to Promote Ciliogenesis

**Figure 3:**
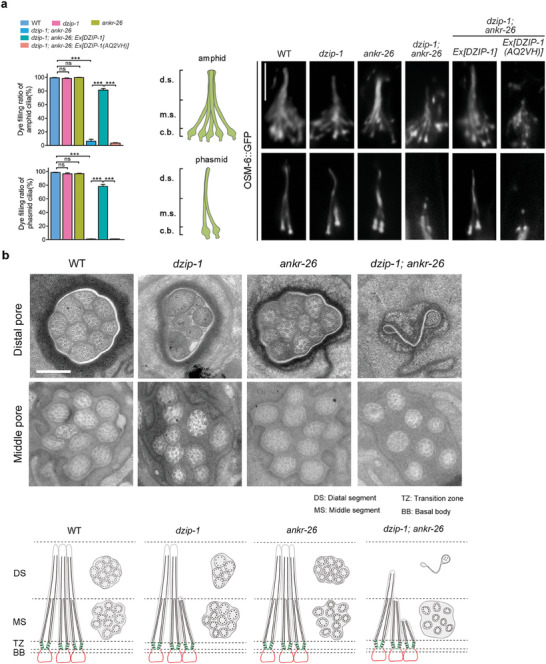
Genetic interaction between DZIP‐1 and ANKR‐26 cooperatively supports ciliogenesis in *C. elegans*. A) The role of DZIP‐1 in ciliogenesis. Left panel: The dye‐filling assay showed that ciliogenesis is impaired in *dzip‐1; ankr‐26* double mutants, but not in *dzip‐1* or *ankr‐26* single mutants. Dye‐filling defect in *dzip‐1; ankr‐26* double mutants can be rescued by introducing the wild‐type sequence of DZIP‐1, but not the sequence contain ARPKD mutations. Middle panel: Diagrams of amphid and phasmid cilia in *C. elegans*. d. s. indicates distal segment, m. s. indicates middle segment, c. b. indicates ciliary base. Right panel: Representative images of cilia labeled with IFT‐B protein OSM‐6::GFP. In *dzip‐1* or *ankr‐26* single mutants, cilia appeared normal, while in *dzip‐1; ankr‐26* double mutants, cilia were significantly truncated. Overexpression of WT DZIP‐1 DNA sequence significantly restored cilia defects in *dzip‐1; ankr‐26* double mutants, whereas DZIP‐1(AQ2VH) mutant sequence did not. Data are presented as the mean ± SEM (*n* > 300 for each genotype from three independent experiments). ***P < 0.001 (Fisher's exact t‐test). Scale bars: 5 µm. B) Transmission electron microscopy (TEM) images of amphid channel cilia in WT, *dzip‐1, ankr‐26*, and *dzip‐1; ankr‐26* mutants. Each amphid channel contains 10 cilia in WT. While the ciliary structures in *ankr‐26* single mutants appeared normal, *dzip‐1* single mutants showed mild defects with partial loss of distal segments. However, in *dzip‐1; ankr‐26* double mutants, the cilia exhibited complete loss of distal segments and partial loss of middle segments. Scale Bars: 200 nm.

We used the Dye‐filling assay and the IFT‐B component OSM‐6/IFT52::GFP to examine cilia integrity in *C. elegans*. The Dye‐filling assay showed that both amphid and phasmid cilia in *dzip‐1* null mutants took up dye normally, indicating no apparent ciliogenesis defects. This was supported by assessing the localization of the IFT‐B component OSM‐6 (the ortholog of human IFT52) (**Figure** **3**a). We then investigated whether *dzip‐1* genetically interacts with *ankr‐26* or *talp‐3*, as their encoded proteins likely form a subcomplex to build the ciliary gate. We found that *dzip‐1; ankr‐26* double mutants, but not *dzip‐1; talp‐3* double mutants, showed synergistic dye‐filling defects in both amphid and phasmid cilia (Figure 3a; Figure S3a, Supporting Information). Cilia morphology examination using GFP‐tagged OSM‐6 and the axonemal tubulin marker mCherry‐tagged TBB‐4 showed that cilia were severely truncated with distal segments absent in *dzip‐1; ankr‐26* double mutants (Figure 3a; Figure S3b, Supporting Information). Cilia defects in *dzip‐1; ankr‐26* double mutants were rescued by the introduction of wild‐type DZIP‐1 protein but not DZIP‐1 carrying ARPKD variants (DZIP‐1(AQ2VH)) (Figure 3a). We further performed transmission electron microscopy (TEM) analysis, which showed a lack of distal segments and partial loss of middle segments in cilia of *dzip‐1; ankr‐26* double mutants (Figure 3b). Notably, although we did not observe ciliary defects in *dzip‐1* single mutants with dye‐filling and fluorescence assays, TEM results indeed showed that partial distal segment cilia loss occurs in *dzip‐1* null cilia, suggesting that DZIP‐1 alone play subtle role in ciliogenesis. Taken together, our results indicate that DZIP‐1 and ANKR‐26 interact genetically to assemble cilia in *C. elegans*.

### 2.6 The DZIP‐1‐ANKR‐26 Module is Vital for Cilia Gating

**Figure 4:**
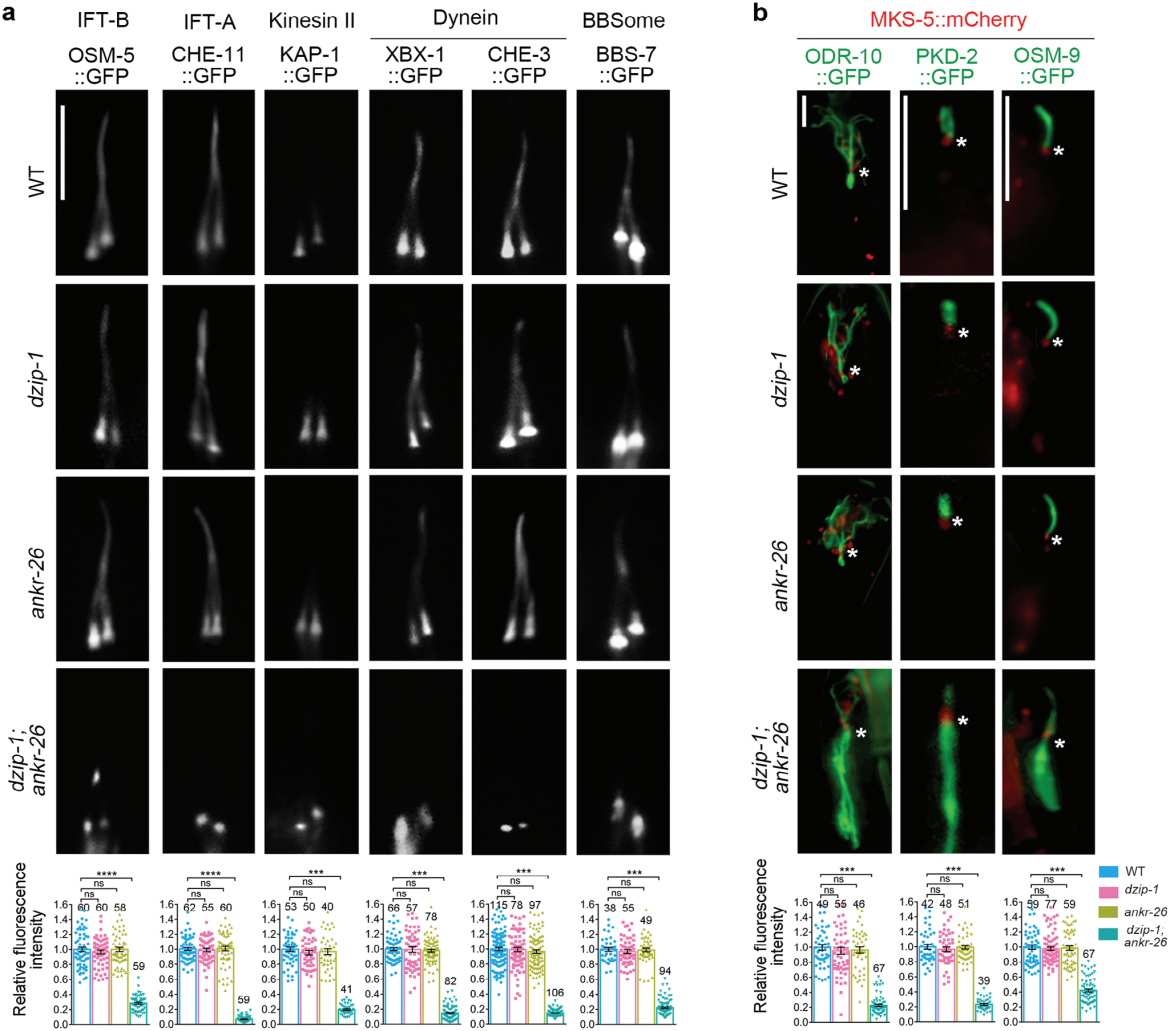
The localization of IFT and ciliary receptors is compromised in *dzip‐1; ankr‐26* double mutants. A) The localization of IFT components (Upper panel) and the quantitation of the relative fluorescence intensity (Lower panel) in the indicated genetic backgrounds. To assess the effects of mutants, a series of GFP‐fused IFT markers from WT were introduced into *dzip‐1* single, *ankr‐26* single, or *dzip‐1; ankr‐26* double mutants, after which their ciliary localization signals were analyzed. The ciliary entry of all IFT proteins is severely compromised in *dzip‐1; ankr‐26* double mutants, but not in *dzip‐1* or *ankr‐26* single mutant. IFT‐A component CHE‐11::GFP, IFT‐A‐associated kinesin‐II subunit KAP‐1::GFP, BBSome protein BBS‐7::GFP, dynein light chain XBX‐1::GFP and heavy chain CHE‐3::GFP were completely restricted to entry the ciliary compartment, whereas IFT‐B component OSM‐5::GFP was able to entry the ciliary compartment and accumulate at the tip of the residual cilia. Scale bars: 5 µm. B) The localization of ciliary membrane proteins ODR‐10, PKD‐2, and OSM‐9 was examined in the specified genetic backgrounds. In *dzip‐1; ankr‐26* double mutants, GFP‐tagged ODR‐10, PKD‐2, and OSM‐9 were found to have abnormal accumulation in the dendrites compared to either single mutants. Lower panel shows the quantitation of the relative GFP fluorescence intensity within cilia in the indicated genetic backgrounds. White asterisks indicate the transition zone marked by MKS‐5::mCherry. The number of cilia analyzed for each genotype is indicated in bars. Scale bars: 5 µm.

TFs are a central part of the ciliary gate essential for regulating the ciliary import of both soluble IFT machineries and membrane receptors.^[^ ^15^, ^23^ ^]^ In order to investigate the involvement of DZIP‐1 and ANKR‐26 in ciliary gating, we introduced a series of GFP‐fused IFT markers and membrane proteins from WT into *dzip‐1* single, *ankr‐26* single, and *dzip‐1; ankr‐26* double mutants, then analyzed their ciliary localization signals. In *dzip‐1* and *ankr‐26* single mutants, the ciliary localization of all ciliogenic proteins examined appears to be comparable to the wild type (**Figure** **4**a,b). Remarkably, in *dzip‐1; ankr‐26* double mutants, the IFT‐B component OSM‐5 (the ortholog of human IFT88) abnormally accumulates at the tip of truncated cilia while other IFT components including IFT‐A subunit CHE‐11 (the ortholog of human IFT140), the BBSome component BBS‐7, kinesin‐II motor KAP‐1, dynein motor light chain XBX‐1 and heavy chain CHE‐3 fails to enter cilia (Figure 4a; Figure S4, Supporting Information). Consistent with gating defects of soluble ciliogenic proteins, the localization of membrane proteins, including three sensory receptors (PKD‐2,^[^ ^30^ ^]^ OSM‐9,^[^ ^31^ ^]^ and ODR‐10^[^ ^32^ ^]^) and the small GTPase ARL‐13, was also compromised in *dzip‐1; ankr‐26* double mutants, displaying mislocalized signals below the ciliary base (Figure 4b; Figure S4a, Supporting Information). The phenotypes observed in the double mutants are similar to the classical gating defects observed in *dyf‐19* mutants.^[^ ^15^ ^]^ These observations support the conclusion that DZIP‐1 and ANKR‐26 genetically interact to play a crucial role in cilia gating.

### 2.7 DZIP‐1 Collaborates with ANKR‐26 to Establish a Functional Cilia Gate

**Figure 5:**
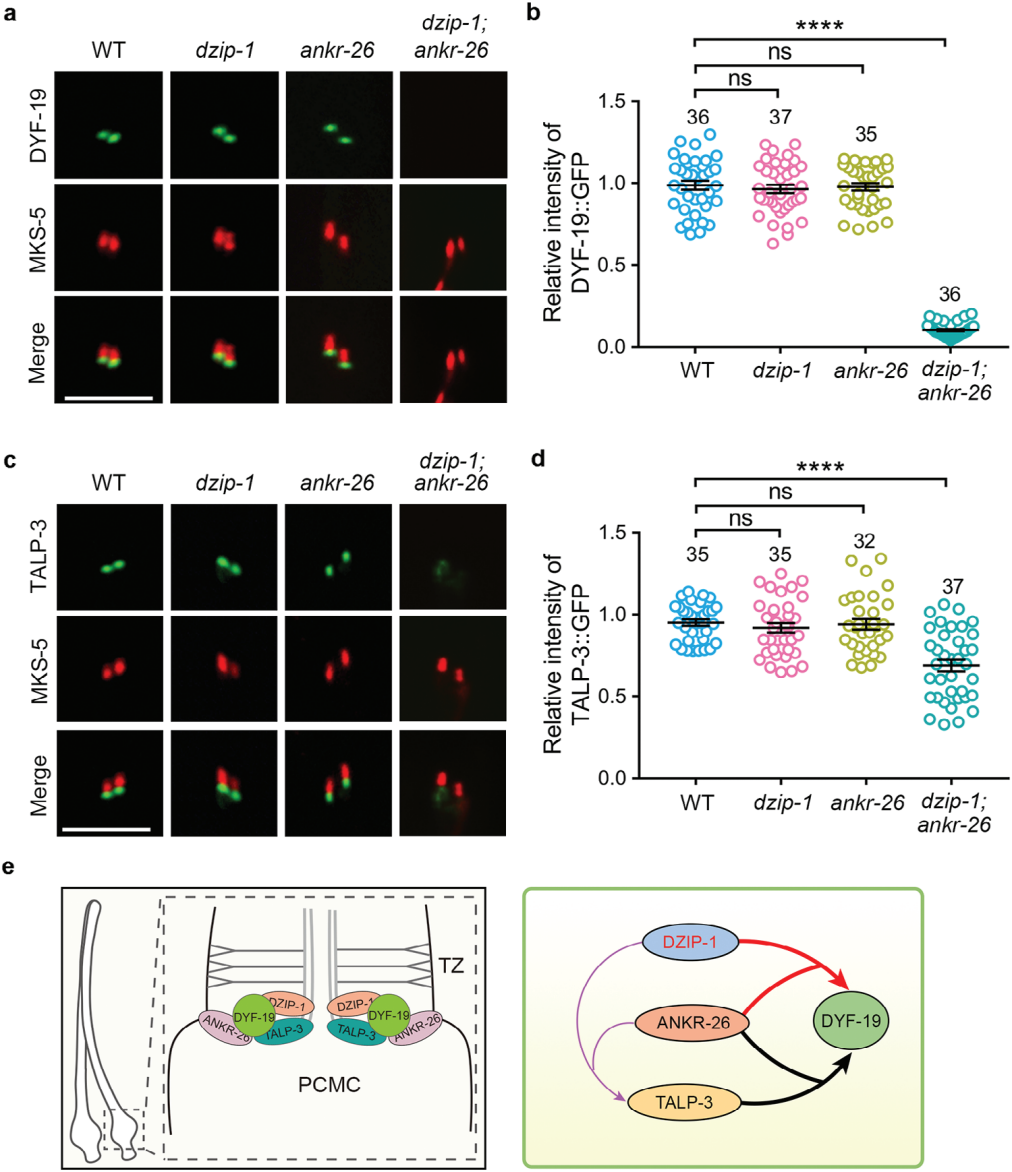
DZIP‐1 and ANKR‐26 cooperate to recruit transition fiber protein DYF‐19/FBF1. A) The transition fiber localization of DYF‐19 was lost in *dzip‐1; ankr‐26* double mutant. B) Relative fluorescence intensity of DYF‐19 was quantified in the indicated genetic background. C) The signal of GFP‐tagged TALP‐3 is diminished and dispersed in PCMC region of *dzip‐1; ankr‐26* double mutants. D) Relative fluorescence intensity of TALP‐3 were quantified in the indicated genetic background. E) Models show the association between DZIP‐1 and other TFs‐related components. Left panel: The spatial relationship among DZIP‐1, ANKR‐26, DYF‐19, and TALP‐3. Right panel: Hierarchical recruitment among DZIP‐1, ANKR‐26, DYF‐19, and TALP‐3. All data are presented as the mean ± SEM (The number of cilia analyzed for each genotype is indicated in bars), Significant differences were identified by Mann‐Whitney test. ns, *P* > 0.05; ****P < 0.0001. Scale bars = 5 µm.

The classical cilia gating defects of *dzip‐1; ankr‐26* double intrigue us to assess the integrity of the ciliary gate. We previously reported DYF‐19 and TALP‐3 are both key players for establishing TFs as a functional gate.^[^ ^15^, ^23^ ^]^ In *dzip‐1* and *ankr‐26* single mutants, the localization of DYF‐19 and TALP‐3 was normal. However, in *dzip‐1; ankr‐26* double mutants, the TF signal of DYF‐19 was lost completely (**Figure** **5**a,b), and the TF signal of TALP‐3 was significantly reduced and dispersed in the PCMC region (Figure 5c,d). These findings indicate that DZIP‐1 and ANKR‐26 work together to recruit DYF‐19 and TALP‐3 to TFs. Based on our previous results demonstrating that the ANKR‐26‐TALP‐3 module is required for TF recruitment of DYF‐19, and there is no genetic interaction between TALP‐3 and DZIP‐1 (Figure S3a, Supporting Information), we propose a hierarchical assembly pathway for TF assembly in *C. elegans* (Figure 5e): DZIP‐1 and ANKR‐26 serve as the core to recruit all other TF components to establish a functional cilia gate.

### 2.8 Spatial Relationship Between DZIP1L and Other TF Proteins in Mammalian Cilia

In mammals, ciliary TFs originate from distal appendages (DAs) of mother centrioles. Consistent with the exclusive TF localization for DZIP‐1, our super‐resolution structured illumination (SIM) microscopy observation revealed that human DZIP1L asymmetrically localizes to the mother centriole right above the subdistal appendages labelled by ODF2 in retinal pigment epithelial (RPE) cells (Figure S5a, Supporting Information). It exhibits a “two dots” pattern on a similar longitudinal plane as TF components FBF1 and ANKRD26, supporting its association with DA/TF. However, the distance between DZIP1L's two dots is notably smaller than that of FBF1 or ANKRD26's two dots (Figure S5b, Supporting Information), suggesting DZIP1L is positioned closer to the centriolar wall. Consistently, in top views, DZIP1L forms a ring with a diameter smaller than that of FBF1 and ANKRD26, yet similar to that formed by TALPID3 (Figure S5b–e, Supporting Information). Nonetheless, the rings formed by DZIP1L partially overlapped with those of FBF1 and ANKRD26.

**Figure 6:**
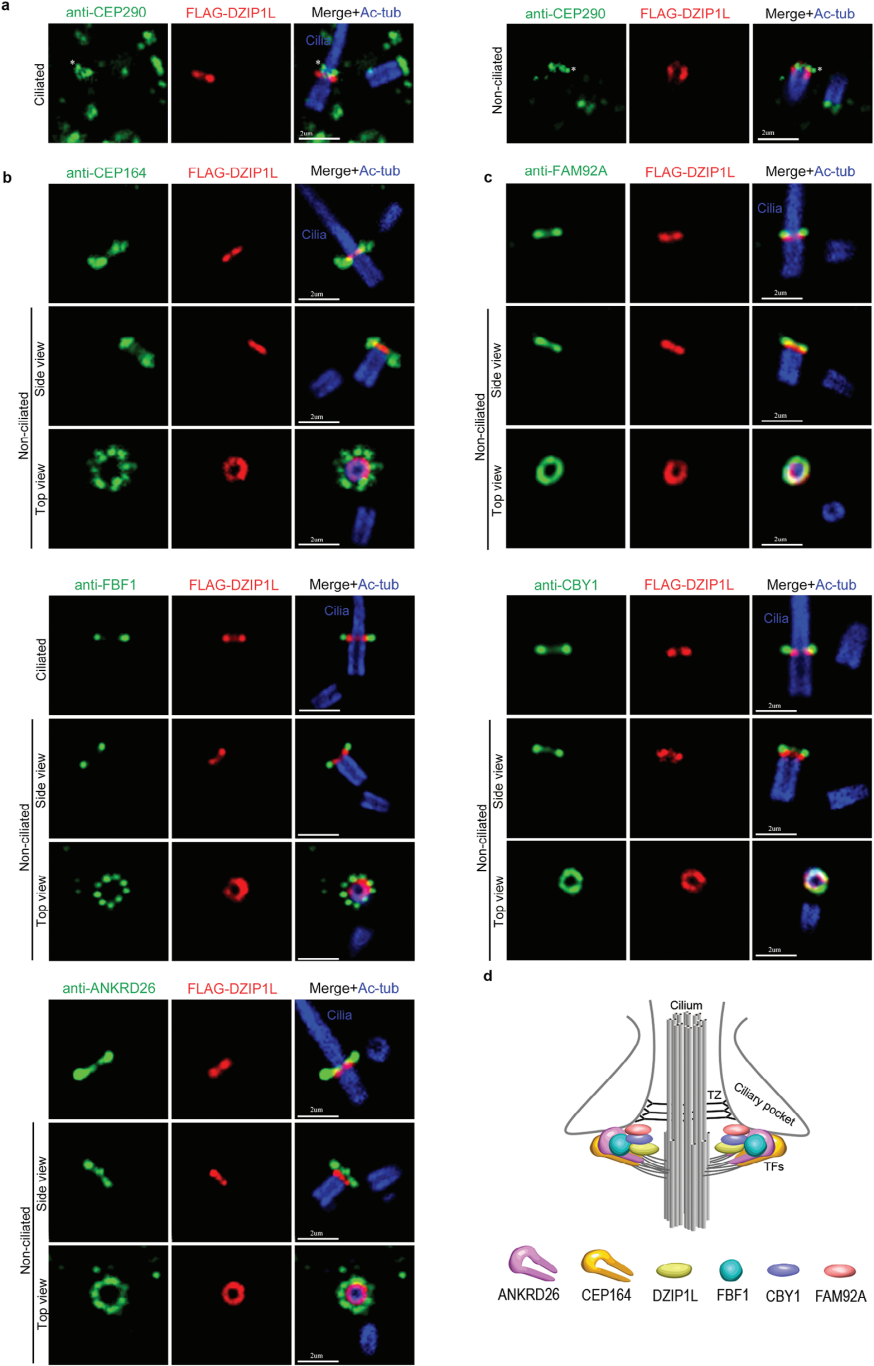
The precise localization of DZIP1L in human RPE cells. Spatial localization of DZIP1L was examined using a combination of Ultrastructure expansion microscopy (ExM) and confocal microscopy. As our DZIP1L polyclonal antibody is derived from rabbits, and most of our antibodies for TF and TZ markers are also polyclonal antibodies from rabbit, co‐labeling them with antibodies is not feasible. To assess their co‐localization, we employed transgenic FLAG‐DZIP1L and utilized a mouse monoclonal FLAG antibody (IgG1 isotype) to label DZIP1L. While cilia were marked using anti‐Ac‐tubulin, a mouse monoclonal antibody with IgG2b isotype. A) FLAG‐DZIP1L was localized beneath the classical TZ component CEP290 in both ciliated and non‐ciliated cells. Asterisks indicate CEP290 signal at the distal of the mother centriole or basal body. B) FLAG‐DZIP1L was positioned at a comparable longitudinal level as TF proteins CEP164 and FBF1, slightly lower than TF protein ANKRD26. FLAG‐DZIP1L exhibited a toroidal organization, with a smaller diameter than that of CEP164, FBF1, and ANKRD26. C) FLAG‐DZIP1L were situated slightly lower than TF‐TZ interface proteins FAM92A and CBY1, with a toroid diameter smaller than that of CBY and FAM92A. D) Cartoon illustrating the precise spatial localization of DZIP1L at cilia base. DZIP1L localizes between TFs and TZ, residing within the space between the centriole wall and TFs, alongside CBY1 and FAM92A, forming a distinct category of TF‐associated proteins. Scale bars: 2 µm.

Given that DZIP1L was previously reported as a transition zone protein in mammals,^[^ ^26^, ^27^ ^]^ its precise localization as either TF‐associated or TZ protein required further elucidation. To address this, we employed a combination of ultrastructure expansion microscopy (U‐ExM) and confocal techniques to carefully examine the spatial relationship of DZIP1L with respect to TF or TZ proteins. Our results clearly showed that in both ciliated and non‐ciliated human RPE cells, DZIP1L resides below the classical TZ component CEP290 (**Figure** **6**a). Co‐labeling DZIP1L with TF markers, including CEP164, FBF1 and ANKRD26, indicated it closer association with TFs, positioning nearer to the centriole wall compared to CEP164, FBF1, and ANKRD26 (Figure 6b; Videos S1–S4, Supporting Information). Given previous reports of DZIP1L's interaction with the CBY‐FAM92 complex,^[^ ^27^, ^30^, ^31^ ^]^ which is associated with the top face of TFs^[^ ^32^, ^33^ ^]^ and plays critical roles in ciliary budding,^[^ ^27^, ^30^, ^34^ ^]^ we further co‐labelled DZIP1L with CBY and FAM92, and observed that DZIP1L is positioned slightly beneath CBY and FAM92, yet remains closer to the centriole wall than CBY and FAM92 (Figure 6c).

Collectively, our results indicate that DZIP1L does not adhere to the conventional structure of a classical TZ component. Instead, it localizes between TFs and TZ, residing within the space between the centriole wall and TFs, alongside CBY and FAM92A, forming a distinct category of TF‐associated proteins (Figure 6e).

### 2.9 Conserved Role for the Mammalian DZIP1L‐ANKRD26 Module in Cilia Gating

**Figure 7:**
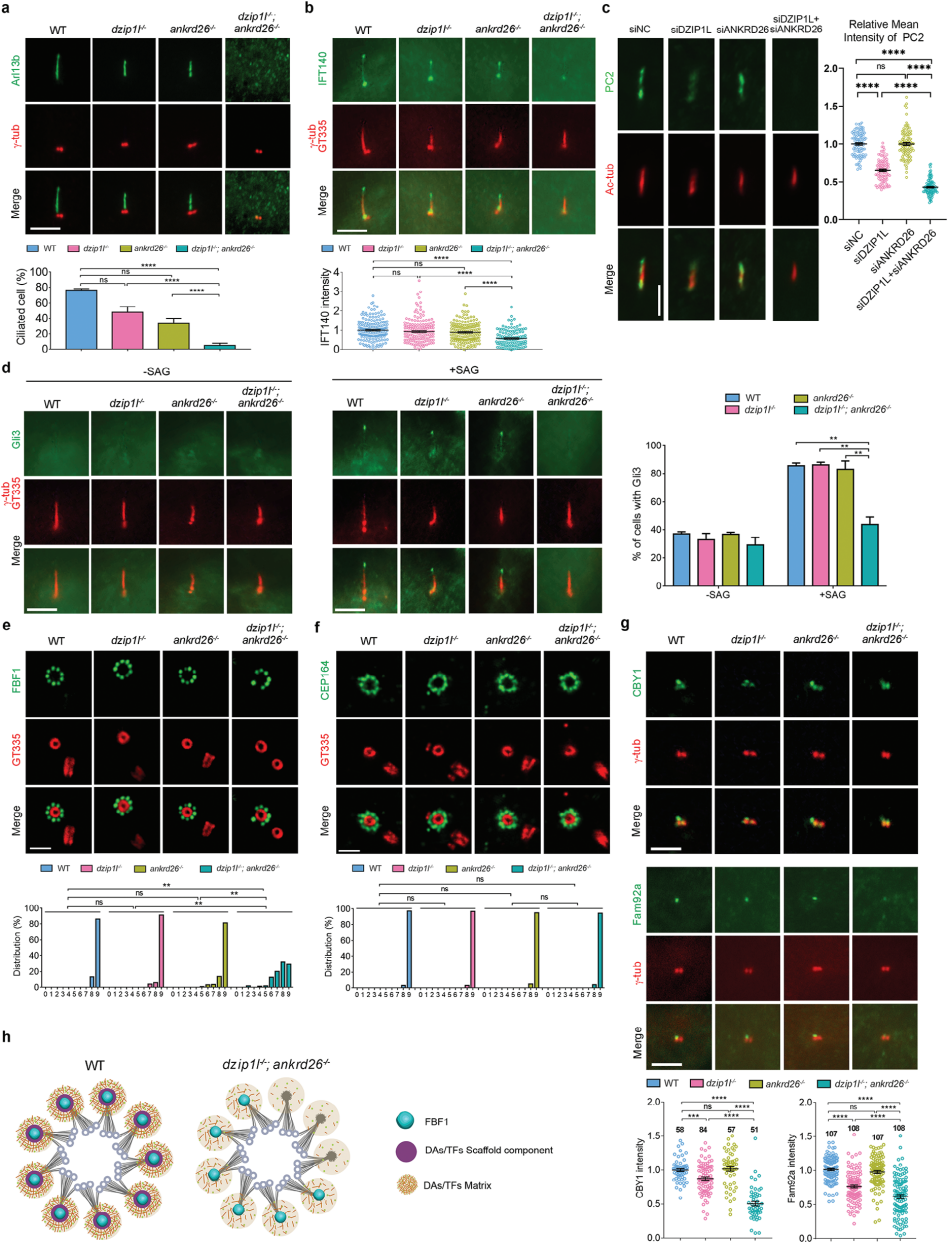
Conserved role of DZIP1L‐ANKRD26 module in cilia gating in human RPE cells. A) The genetic interaction between DZIP1L and ANKRD26 in cilia formation is conserved in mammalian cells. The knockouts of either DZIP1L or ANKRD26 led to a moderate decrease in ciliation ratio. However, when both DZIP1L and ANKRD26 were knocked out, cilia formation was nearly completely blocked. The ciliation ratio was shown in the lower panel. Data are presented as mean ± SEM. *n* > 300 cells from three independent experiments. Significant differences were determined by two tailed t‐test analysis. Scale bars: 5µm. B) Ciliary entry of the IFT component IFT140 was compromised in double mutants of DZIP1L and ANKRD26, compared to either of single mutants. Lower panel: Quantification of the relative intensity of IFT140 in the specified genetic background. ns, *P* > 0.05; **, *P* < 0.01 (Fisher's exact test). Scale bars: 5µm. C) DZIP1L and ANKRD26 double‐knockdown cells showed significantly reduced ciliary entry of ADPKD protein polycystin‐2 (PC2), compared to cells with a single knockdown. The quantification of relative intensity of PC2 was showed on the right. D) Gli3 localization was analyzed in indicated genetic background RPE‐1 cells treated without (‐SAG) or with (+SAG) SAG. Right panel: Percentage of ciliated cells with Gli3 were quantified. *n* = 300 cells from three independent experiments were statistically analyzed. Data are presented as the mean ± SEM. ns, *P* > 0.05; **, *P* < 0.01 (Fisher's exact test). Scale bars: 5µm. E,F) The localization of FBF1 and CEP164 was examined using Ultrastructure Expansion Microscopy (U‐ExM) in the specified genetic background. (E) FBF1 organization at distal appendages was compromised in *dzip1l; ankrd26* double mutants. Upper panel: Confocal images of expanded centrioles stained with FBF1. Cells were stained with GT335 (red) and FBF1 (green dots). Lower panel: The distribution of the number of dots formed by FBF1 in the indicated genetic background. ns, P > 0.05; **, P < 0.01 (Mann–Whitney test). Scale bar: 2µm. (F) The localization of Cep164 is normal in *dzip1l; ankrd26* double mutants. Upper panel: Confocal images of expanded centrioles stained with CEP164. Cells were stained for GT335 (red) and CEP164 (green dots). Lower panel: The distribution of the number of dots formed by CEP164 in the indicated genetic background. ns, *P* > 0.05; Scale bar: 2µm. G) The localization of CBY1 and Fam92a to the distal centriole was significantly impaired in *ankrd26* and *dzip1l* double mutants, whereas either single mutant only exhibited slight defects in their localization. Upper panel: Representative images of the localization of CBY1. Cells were stained for γ‐tub (red) and CBY1 (green). Middle panel: Representative images of the localization of Fam92a. Cells were stained for γ‐tub (red) and Fam92a (green). Lower panel: The relative fluorescence intensity of CBY1 and Fam92a. Data are presented as the mean ± SEM (The number of cells examined is indicated above each dataset). Significant differences were identified by Mann‐Whitney test. ns, *P* > 0.05; ***, *P* < 0.001; ****, *P* < 0.0001. Scale bars: 5 µm. H) A prospective model of the role of DZIP1L and ANKRD26 in the formation of transition fiber matrixes, which serve as the functional site for selective cilia gating. The transition fiber matrix encircles FBF1, the key functional protein in TFs, and houses various functional proteins such as IFT, receptors, Cby, and Fam92. Deletion of DZIP1L and ANKRD26 disrupts the proper localization of FBF1 and compromises the integrity of the matrix, leading to defects in ciliogenesis.

Our interest in understanding the function of mammalian DZIP1L led us to investigate its genetic interaction with ANKRD26 in mammalian cells using CRISPR‐Cas9 gene editing technique (Figure S6a–d, Supporting Information). Different than what we observed in *C. elegans*, in human RPE cells, knockout of either DZIP1L or ANKRD26 individually already resulted in moderate reduction in ciliation ratio, while the ciliation was almost completely blocked in the double knockout mutants (**Figure** **7**a). Consistently, the ciliary entry of IFT140 was significantly disrupted in remaining cilia of *DZIP1L^−/−^; ANKRD26^−/−^* double mutant cells (Figure 7b). To further explore the role of DZIP1L in cilia gating, we examined PKD2, and hedgehog signaling molecular GLI3. As expected, a significant compromise in the ciliary entry of PKD2 and GLI3 were observed in remaining cilia of the double mutant cells, more so than in either single mutant cells (Figure 7c,d). These findings suggest that there is a conserved genetic interaction between DZIP1L and ANKRD26 in regulating cilia gating in mammalian cilia.

Continuing our exploration of the role of DZIP1L in cilia gating, we examined the impact of DZIP1L deficiency on the organization of TF architecture in cilia of human RPE cells. Under the confocal microscopy, no apparent defects were observed in the localization of FBF1 and Cep164 in either single or double knockout cells (Figure S7, Supporting Information). Intriguingly, by combining U‐ExM and confocal, we observed that the TF anchoring of FBF1, a key functional player involved in TF‐mediated cilia gating,^[^ ^15^ ^]^ is significantly compromised in *DZIP1L^−/−^; ANKRD26^−/−^* double knockout cells (Figure 7e). However, the localization of structural component CEP164 did not show any abnormalities in either single or double knockout cells (Figure 7f). In line with these finding, the double knockout cells showed accelerated impaired localization of TF‐associated functional components, including CBY1^[^ ^35^ ^]^ and FAM92A^[^ ^32^, ^36^, ^37^ ^]^ (Figure 7g). Of note, many ciliogenic proteins examined here already show compromised, although subtle, ciliary targeting in *DZIP1L^−/‐^* single knockout cells, suggesting mammalian DZIP1L plays a more critical role than its worm counterparts in the context of cilia gating.

Taken together, we demonstrated that the coordinate role of DZIP1L and ANKRD26 in TF architecture and function is conserved in mammalian cells. We propose that TF matrixes are present between individual fibers of TFs, consisting of various proteins such as IFT protein, ciliary receptors, CBY, FAM92, et al., and serve as the functional sites for selective cilia gating. The organization of TF matrixes is critically dependent on FBF1. Deletion of DZIP1L and ANKRD26 disrupts the proper localization of FBF1 and compromises the integrity of the matrix (Figure 7h), leading to defects in cilia gating.

## 3 Discussion

This report presents evidence that the ARPKD protein DZIP1L plays a crucial role in defining TFs as the ciliary gate. We demonstrated that the double knockout of DZIP1L and the TF protein ANKRD26 leads to a significant worsening of defects in cilia gating due to disrupted TF integrity in mammalian cells (Figure 7a–f). Given that ARPDK is genetically inherited kidney diseases that exhibits varying degrees of severity among individuals, several genetic modifiers have been proposed to account for the variability.^[^ ^33^ ^]^ Our findings suggest that ANKRD26, and likely other TF components/regulators, may act as genetic modifiers of ARPKD and indicate a direct association between TF function and the pathogenesis of ciliopathies beyond PKDs.

Previously, DZIP1L was implicated as a transition zone protein in mammalian cells, although their data showing partial co‐localization of DZIP1L with both TF protein CEP164 and TZ proteins.^[^ ^26^, ^27^ ^]^ TFs and the TZ are closely adjacent structures, spanning ≈1 µm in length in most mammalian cilia. The lower resolution in those studies hindered the accurate evaluation of sub‐ciliary localization of DZIP1L within the context of TFs or the TZ. Here, employing a combination of U‐ExM and confocal techniques, we clearly demonstrated that DZIP1L is a TF‐associated protein. It localizes below the classical TZ protein CEP290, slightly lower than the TF‐TZ interface proteins CBY and FAM92A, and at a similar longitudinal level with TF proteins, residing within the space between TF proteins and the centriole wall. These observations are consistent with the recent report by Garden et al.,^[^ ^22^, ^33^, ^38^ ^]^ where they demonstrated that DZIP1, CBY and FAM92A are part of the distal appendage/transition fiber‐associated proteins, and their localization depends on CEP83, a fundamental member of transition fibers. Notably, considering that earlier studies utilized antibodies to mark DZIP1L, whereas our study employs Flag‐tagged DZIP1L, there may be slight variations in localization, thus the exclusion of TZ localization of DZIP1L cannot be guaranteed.

In human cells, DZIP1L localizes between TF and TZ, whereas its homolog, DZIP‐1, in *C. elegans*, exhibits near co‐localization with TF‐related proteins. The specific basal body structure in *C. elegans* likely contributes to these slight localization variations. Previous reports indicate that in *C. elegans*, the basal body degenerates post‐cilia assembly, resulting in flared microtubule doublets and the absence of most core basal body components,^[^ ^39^, ^40^ ^]^ leaving only a subset of TF‐associated proteins, including HYLS‐1, FBF‐1, TALP‐3, ANKR‐26, and DZIP‐1. The presence of flared microtubules may alter the proximity and organization between TF and TZ, potentially complicating the distinction of the space between TF and TZ.

In *Drosophila*, the sole homolog of DZIP1/DZIP1L, interacts with Cby and Fam92, and exhibits co‐localization with TZ proteins MKS1 and MKS6, playing crucial roles in TZ assembly.^[^ ^30^, ^31^ ^]^ However, it is important to note that they do not belong to either the NPHP module or MKS module, signifying that they do not serve as classical structural components for TZ. Instead, their potential role lies in regulating TZ assembly by influencing the initiation of ciliogenesis through the modulation of early ciliary membrane formation.^[^ ^30^ ^]^ This difference in localization maybe an evolutionary divergence between *Drosophila* and other species. Interestingly, besides its TZ localization, *Drosophila* DZIP1 also localizes between TFs and TZ, particularly in sensory cilia.^[^ ^30^ ^]^ And our unpublished data showed that DZIP1 is required for the proper localization of FBF1 in sensory cilia in *Drosophila*, suggesting a conserved role in maintaining TF integrity. According to our data and the available literature, we propose that DZIP1L does not conform to the conventional structure of a classical TZ component. Rather, it positions between TFs and TZ, within the space between TF's outer head and the centriole. Alongside Cby and Fam92, it forms a category of TF‐associated proteins, and may potentially interact with some TZ proteins due to their close sub‐ciliary localization.

Selective import of ciliogenic proteins is crucial for making the cilium a distinct sensory entity.^[^ ^41^, ^42^ ^]^ TFs have been identified as a critical component of the ciliary gate, which is the first visible physical barrier between the cytoplasm and the ciliary lumen. The amorphous space (referred here as TF matrixes) between individual fibers of TFs, possessing a higher concentration of ciliary proteins, including IFT particles, has been suggested to be important sites for this selective cilia import.^[^ ^22^ ^]^ Although TFs’ anatomical structure was described more than five decades ago,^[^ ^10^ ^]^ the structure basis of TF blades and the organization of TF matrixes remain poorly characterized. Recent studies have made significant progress in identifying TF components, over 10 TF localized proteins have been identified in mammals, including CEP83, SCLT1, CEP164, CEP89, FBF1, ANKRD26, TTBK2, and so on.^[^ ^12^, ^13^, ^14^, ^15^, ^16^, ^17^, ^18^ ^]^ Among them, FBF1 is the first identified core functional, but not structural, component of TFs.^[^ ^22^ ^]^ FBF1 deficiency impairs gating function, but not the localization of other TF proteins.^[^ ^15^, ^23^ ^]^ Except for DZIP1L studied here, our previous study has also identified TALPID3 as a TF‐associated protein that work in coordination with ANKRD26 to ensure the proper TF localization of FBF1.^[^ ^23^ ^]^ However, it is important to note that this regulation seems to be specific to FBF1 localization and does not affect the localization of other proteins within TF blades. We propose that FBF1 is a TF protein that plays a critical role in organizing the TF matrix, the highly conserved TF‐related protein DZIP1L, TALPID3 and ANKRD26, are likely to be involved as components or regulators of the proteinaceous matrixes between the fibers. To maintain the integrity of TF matrixes, coordination is required between proteins located on TF blades and centriole wall.

Interestingly, while TFs are widely recognized as structures derived from distal appendages of the mother centriole in mammalian cells,^[^ ^10^ ^]^ it is noteworthy that *C. elegans* centrioles lack distal appendages yet still possess TF‐like formations at the base of their cilia.^[^ ^43^ ^]^ Correspondingly, the putative structure constituents of TFs, CEP83, SCLT1, CEP89, and CEP164, do not have counterparts in the *C. elegans* genome, nevertheless, the functional components involved in ciliary gating, such as FBF1, ANKRD26, DZIP1L and TALPID3, are conserved between *C. elelgans* and mammals, both in subcellular localization and the regulation of cilia gating. Hence, while the precise structural morphology of TFs may differ among species throughout evolution, the functional subdomain associated with TFs and cilia gating remains evolutionarily conserved, representing a critical aspect of TF‐like structures in the early stages of evolutionary development.

In mammals, *DZIP1L* has a close homolog *DZIP1*, which also plays a role in ciliogenesis.^[^ ^44^ ^]^ Previous RNAi studies have suggested that DZIP1 and DZIP1L have redundant function in the formation of cilia in mammalian cells.^[^ ^45^ ^]^ It is possible that they also have overlapping roles in regulating the architecture and integrity of TFs, which may explain why we observed only moderate defects in the localization of FBF1 in mammalian cells with double knockout of *DZIP1L* and *ANKRD26* (Figure 7e). Intriguingly, *Dzip1* mutant mice either grow to maturity without noticeable abnormalities,^[^ ^44^ ^]^ or only show infertility.^[^ ^46^ ^]^ It would thus be interesting to investigate the functional redundancy and tissue specificity between DIZP1 and DZIP1L in the regulation of cilia gating in future.

The two ARPKD mutations, p.A90V and p.Q91H,^[^ ^26^ ^]^ of DZIP1L locate in the highly conserved DZIP‐like domain. While homozygous *null* mice for *Dzip1* (*Dzip1l^wpywpy/^*) exhibit severe ciliopathy defects, including embryonic lethality and failure to thrive postnatally, ARPKD patients with DZIP1L^A90V^ or DZIP1L^Q91H^ mutations only display mild PKD and hypertension, but are otherwise healthy to adulthood.^[^ ^26^ ^]^ The drastic difference suggests that DZIP1L^A90V/Q91H^ may specifically affect pathways implicated in PKD pathogenesis but have a benign effect on other DZIP1L‐regulated functions in cilia gating. Therefore, it is plausible that the ARPKD variants of DZIP1L may impair the ciliary import of ADPKD protein polycystins but subtly affect cilia gating of other ciliogenic proteins. A common feature of pathogenic mutations in ADPKD is a resulting deficiency in the surface localization of polycystins.^[^ ^47^ ^]^ Understanding how cilia import of PKD proteins is controlled will be crucial in developing therapeutic strategies for PKD diseases.

## 4 Experimental Section

### Caenorhabditis Elegans Strains

Strains used in this study are listed in Table S1 (Supporting Information). N2 worms represented the wild type in all experiments. Worms were cultured and maintained on NGM plates seeded with *Escherichia coli* strain OP50 at 20 °C. For *dzip‐1(C01G5.7)* mutant worms, full length of *c01g5.7* (from ATG to stop codon) was knocked out by FUJIAN sunybiotech co., ltd. using CRISPR‐Cas9 technology according to the design. Standard genetic crossing was used to introduce GFP‐fused transgenes to mutant worms. To ensure that expression levels of GFP markers are comparable between the wild‐type and mutant strains, each GFP transgene from the wild‐type worm was individually introduced into mutant worms via genetic crossing.

### Dye‐Filling Assay

Worms were rinsed into a 1.5 mL EP tube with M9 buffer, centrifuged at low speed for 1 min, and then washed twice with M9 buffer. Then DiI dye was added for the final concentration of 10 µg mL^−1^ and placed on a rotating shaker at room temperature for 1 h. After this, worms were washed three times with M9 buffer and transferred to a dish. Subsequently, the dye filling of worm amphids was observed under the Nikon SMZ18 stereo‐fluorescence microscope, and the dye filling of phasmids was observed and calculated using the Nikon Eclipse Ti2 fluorescence microscope with a 100x objective.

### Transmission Electron Microscopy

Young adult worms were collected with M9 buffer. Replace M9 buffer with 2.5% glutaraldehyde and centrifuged for 1 min at slow speed, and then discard the supernatant, and add 300 µL 2.5% glutaraldehyde again. Then the worms were stored at 4 °C for 24 h. Afterward, 2.5% glutaraldehyde was replaced with 1% osmic acid and worms were stored at 4 °C for 4 h, then they were dehydrated with a series of concentration gradients of ethanol, followed by three washes with 100% acetone, and then permeated with a series of proportional gradients of acetone and EPON812 resin until permeated with pure EPON812 resin. Serial sections (∼70 nm) were collected from worm head and imaged with a transmission electron microscope (Hitachi H‐7650, Hitachi).

### BiFC (Bimolecular Fluorescence Complementation) Assay

BiFC assay was performed as previously described.^[^ ^48^ ^]^ VN173 and VC155, which are complementary fragments of Venus, were fused to DNA sequences of the relevant genes under control of the ciliated cell‐specific promoter *arl‐13*. Vector mixture (30 ng uL^−1^ for each vector, < 200 ng uL^−1^ for total mixture) was injected into the gonads of N2 animals together with a dominant co‐injection marker pRF4 (rol‐6(su1006)) and a TZ marker MKS‐5::mCherry to generate extrachromosomal transgenic lines. The following BiFC pairs were used in this study: DZIP‐1::VC155 and DYF‐19::VN173, DZIP‐1::VC155 and ANKR‐26::VN173, DZIP‐1::VC155 and TALP‐3::VN173, TALP‐3::VC and DYF‐19::VN, TALP‐3::VC155 and ANKR‐26::VN173, DYF‐19::VC155 and ANKR‐26::VN173. Fluorescence images were visualized and captured using the YFP filter under a Nikon Eclipse Ti2 fluorescence microscope.

### Immunofluorescence and Imaging

Agarose pads were prepared at a concentration of 4% and was dropped onto the slide and then flattened with the slide. Subsequently, add a drop of levamisole at a concentration of 20 mm, and worms were then picked into levamisole for anesthesia. Images were acquired using a Nikon Eclipse Ti2 fluorescence microscope. Worms at L4 stage or young adult were used to count dye filling ratio, and statistical analysis of fluorescence intensity was performed using the software that came with NIS‐Elements.

Cells grown on glass coverslips were washed twice with PBS, fixed at −20 °C with cold methanol for 15min, and washed twice with PBS. Cells were then blocked in PBS containing 3% BSA and sequentially incubated with primary and secondary antibodies. Images were acquired using a Nikon Ti2 fluorescence microscope with a 100× (1.4 NA) oil‐immersion objective, or a Leica Stellaris 5 or Abberior STEDYCON (Abberior Instruments GmbH, Göttingen, Germany) confocal microscope with a 63× (1.4 NA) oil‐immersion objective, or the Delta Vision OMX SR (GE Healthcare) with a 60× (1.42 NA) oil‐immersion objective.

### 3D Reconstruction Video

For 3D movies, confocal images were captured. Centrioles or cilia were scanned in the z‐axial direction with 30‐nm step sizes. Imaging and image processing was done with ImageJ software and then using Imaris software for 3D model reconstruction and video generation.

### Cell Culture and Transfection

Human retinal pigment epithelial (hTERT RPE‐1) were cultured in Dulbecco's modified Eagle's medium (DMEM) supplemented with 10% FBS. To induce cilia formation, cells were starved in serum‐free medium for 24h‐48h. For plasmid transfection in genome editing, Lipofectamine 2000 (Invitrogen) was used following the manufacturer's manual. Flag‐DZIP1L and DZIP1L‐GFP plasmids were transfected via virus delivery. In brief, to produce the virus, HEK293T cells were co‐transfected with the PCDH plasmid containing Flag‐DZIP1L or DZIP1L‐GFP along with lentiviral packaging plasmids psPAX2 and PMD2.G. After 48 h, viral particles were collected and then frozen at −80 °C. Cells were then infected with the virus. To enhance the infection efficiency, selection was performed using G418 (500 µg mL^−1^, added to the culture medium).

### CRISPR‐Cas9 Gene Editing

Guide RNA (gRNA) of DZIP1, DZIP1L and ANKRD26 were designed using an online tool (http://crispor.tefor.net/) and subcloned into pSpCas9 (BB)‐2A‐GFP (px458). RPE1 cells were transfected with gRNA construct for 48 hours and then subjected to flow cytometry to sort single GFP‐positive cells in 96‐well plates, because of a green fluorescent protein (GFP) was fused to Cas9 in px458 plasmid. Single clones were incubated for about 2 weeks. On‐target cleavage in genes was confirmed by Sanger sequencing of PCR products spanning the edited site. Protein localization was confirmed by immunofluorescence imaging. gRNA sequence was as follows:

DZIP1L gRNA#1, 5′‐ TGATCATCTTGCGACGCCGG‐3′;
DZIP1L gRNA#2, 5′‐ CAGTCCATGCTATCATGGCG‐3′;
ANKRD26 gRNA#1, 5′‐GGGTAGCTCACAATCCTCTG‐3′;
ANKRD26 gRNA#2, 5′‐ AAATTCTTGTAACTAGAGTG‐3′;

### Antibodies

The following primary antibodies were used in this study: ANKRD26 (rabbit, GeneTex, GTX128255, 1:500), DZIP1L (rabbit, Proteintech, 17474‐1‐AP, 1:500), FBF1 (rabbit, Proteintech, 11531‐1‐AP, 1:500), ARL13B (rabbit, Proteintech, 17711‐1‐AP, 1:500), KIAA0586/TALPID3 (rabbit, Proteintech, 24421‐1‐AP, 1:500), SCLT1 (rabbit, Proteintech, 14875‐1‐AP, 1:500), CEP164 (rabbit, Proteintech, 22227‐1‐AP, 1:500), CEP89 (rabbit, Abcam, ab204410, 1:500), ODF2 (rabbit, Abcam, ab43840, 1:500), ODF2 (mouse, Abnova, H00004957‐M01, 1:500), γ‐tublin (mouse, Sigma‐Aldrich, T6557, 1:1,000), FAM92A (rabbit, Proteintech, 24803‐1‐AP, 1:500), CBY1 (rabbit, Proteintech, 12239‐1‐AP, 1:500), GFP(mouse, Roche, 11814460001,1:200), IFT140 (rabbit, Proteintech, 17460‐1‐AP, 1:500), GT335(mouse, adipogen life science, A40251903), GLI3 (AF3690, R&D Systems), PC2 (Baltimore Polycystic Kidney Disease (PKD) Research and Clinical Core Center), Ac‐tubulin (mouse, sigma, T7451, 1:300), Flag (mouse, sigma, F1804, 1:300), CEP290 (rabbit, abcam, ab84870).

### Ultrastructure Expansion Microscopy (U‐ExM)

RPE‐1 cells are processed for expansion as previously described.^[^ ^49^ ^]^ Briefly, the cells were cultured on 12 mm coverslips for overnight. Coverslips were then incubated in FA/AA solution (Formaldehyde (FA) and acrylamide (AA) solutions (1.4%FA and 2% AA) prepared with PBS) at 37 °C for 5 h. Prepare the monomer solution (19% sodium acrylate, 0.1% bis‐ acrylamide, and 10% acrylamide) in advance, TEMED and APS (final concentration 0.5% each) were added to the monomer solution and quickly mixed before adding a cover slip. Gelation was allowed to proceed on ice for 5 min, and then the cells were moved to 37 °C in the dark for 1 h. Coverslips with gels were then transferred into denaturation buffer (200 mm SDS, 200 mm NaCl, 50 mm Tris, ddH_2_O double distilled water) for 15 min at room temperature (RT). Subsequently, the gel was removed from the cover slip, replaced with fresh denaturation buffer, and denatured at 95 ° C for 90 min. After denaturation, the gel was immersed in ddH_2_O at room temperature for initial expansion, and water was exchanged at least twice every 30 min. Then, the gel was incubated in ddH2O overnight to allow it to expansion completely. Next, the gel was washed twice in PBS for 15 min to remove excess water, and then incubated with the primary antibody solution. Cells on the gel were incubated with primary antibodies diluted in 2% PBS/BSA for ≈3 h at 37 °C or overnight at 4 °C with gentle shaking. The gel was washed three times in PBS with 0.1% Tween 20 (PBST) and shaken for 10 min. The cells were then incubated with secondary antibody solution diluted in 2% PBS/BSA for 3 h at 37 °C or overnight at 4 °C with gentle shaking. Then the gel was washed 3 times in PBST with shaken for 10 min. Finally, it was placed in a beaker filled with ddH_2_O for final expansion for 30 min, and the water was changed at least twice every 30 min, and then it was completely expanded overnight.

### Quantifications and Statistical Analysis

For categorical data, statistical differences between two samples were analyzed by Fisher's Exact t‐test. For continuous data, statistical differences between two samples were analyzed by Mann‐Whitney test. *P* values > 0.05 were considered to be a nonsignificant difference (ns). *P* values < 0.05 (marked as *); *P* values < 0.01 (marked as **); *P* values < 0.001 (marked as ***) and *P* values < 0.0001 (marked as ****) were considered to be a significant difference.

## Conflict of Interest

The authors declare no conflict of interest.

## Author Contributions

H.C., Z.W., and Z.Y. contributed equally to this work. Q.W. and J.H. initiated and supervised the project. H.C., Z.W., and Z.Y. prepared and carried out most of experiments and data analysis. C.C., Y. Z., and Y. G. assisted the cell experiments. Y.H., Y.W., and Q.W. assisted the *C. elegans* experiments. J.H. and Q.W. drafted the manuscript with the help of K.L., H.C., Z.W., and Z.Y.

## Supporting information

Supporting Information

Supplemental Video 1

Supplemental Video 2

Supplemental Video 3

Supplemental Video 4

## Data Availability

The data that support the findings of this study are available in the supplementary material of this article.
