# Supplementary material for: The ARPKD Protein DZIP1L Regulates Ciliary Protein Entry by Modulating the Architecture and Function of Ciliary Transition Fibers
Source: Adv Sci (Weinh). 2024 Apr 17;11(24):2308820. doi: 10.1002/advs.202308820 (PMC11200010; doi:10.1002/advs.202308820)
Supplement: Supplementary file 1 — Supporting Information [file ADVS-11-2308820-s002.pdf]

ADVANCED SCIENCE
Open Access

Supporting Information

for *Adv. Sci.*, DOI 10.1002/advs.202308820

The ARPKD Protein DZIP1L Regulates Ciliary Protein Entry by Modulating the Architecture and Function of Ciliary Transition Fibers

Huicheng Chen, Zhimao Wu, Ziwei Yan, Chuan Chen, Yingying Zhang, Qiaoling Wang, Yuqing Gao, Kun Ling, Jinghua Hu and Qing Wei**

The ARPKD Protein DZIP1L Regulates Ciliary Protein Entry by Modulating the Architecture and Function of Ciliary Transition Fibers

Huicheng Chen[1, 2, 3, †], Zhimao Wu[3, †], Ziwei Yan[1, 2, †], Chuan Chen[4], Yingying Zhang[3], Qiaoling Wang[5], Yuqing Gao[3], Kun Ling[4], Jinghua Hu[4, *], Qing Wei[3, 6, *]

[1]CAS Key Laboratory of Insect Developmental and Evolutionary Biology, CAS Center for Excellence in Molecular Plant Sciences, Chinese Academy of Sciences, Shanghai 200032, China.
[2]University of Chinese Academy of Sciences, Beijing 100039, China.
[3]Center for Energy Metabolism and Reproduction, Institute of Biomedicine and Biotechnology, Shenzhen Institutes of Advanced Technology, Chinese Academy of Sciences (CAS), Shenzhen 518055, China.
[4]Department of Biochemistry and Molecular Biology, Mayo Clinic, Rochester, MN 55905, USA.
[5]Institute of Medicine and Pharmaceutical Sciences, Zhengzhou University, Zhengzhou 430000, China.
[6]School of Synthetic Biology, Shanxi Key Laboratory of Nucleic Acid Biopesticides, Shanxi University, Taiyuan 030006, China.
†These authors contributed equally to this work.
*Corresponding author. Email: hu.jinghua@mayo.edu (J. H.); qing.wei@siat.ac.cn (Q. W.)

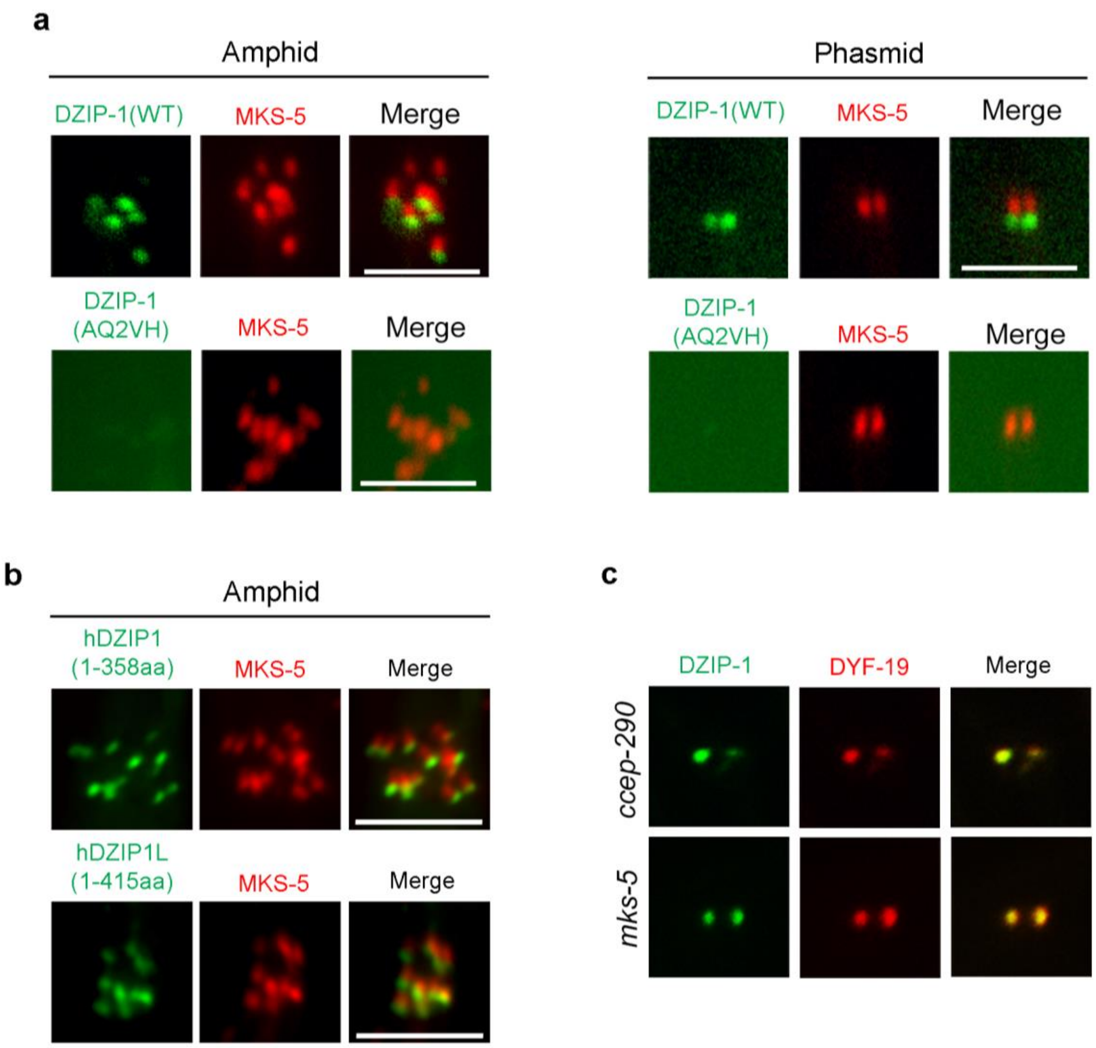

Figure S1. The localization of DZIP-1 in *C. elegans*. (A) DZIP-1 localizes to the base of amphid cilia in *C. elegans*, and ARPKD associated mutations (p.A90V and p.Q91H) affects the localization. (B) N-terminal sequences of human DZIP1 (1-358aa) and DZIP1L (1-415aa) targets to the ciliary base in amphid of *C. elegans*. (C) TZ components (MKS-5 and CEP290) are not required for DZIP-1 localization at ciliary base.

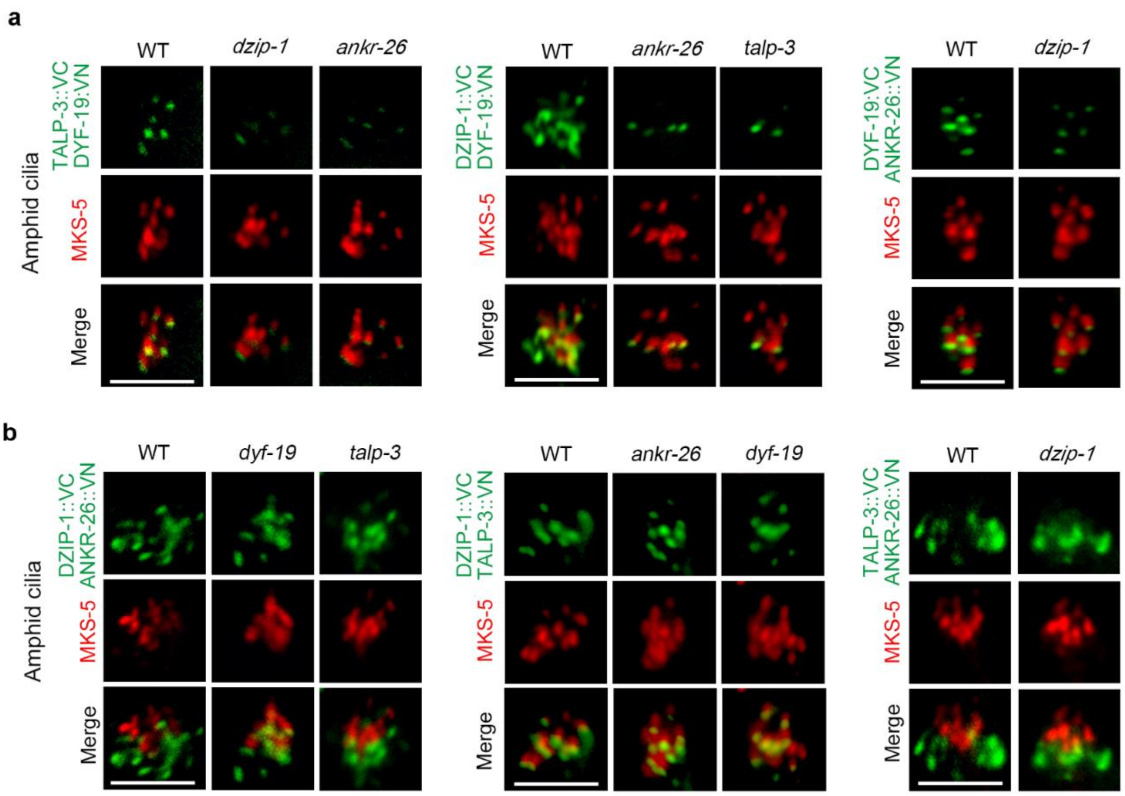

Figure S2. In vivo association between DZIP-1 and other TFs components in amphid cilia of *C. elegans*. (A) BiFC signals were observed between DYF-19 and other TF components in amphid cilia of worms with indicated genetic background. The fluorescence complementation signal between DYF-19 and TALP-3 was disrupted in *dzip-1* or *ankr-26* single mutants, similarly, the fluorescence complementation signal between DYF-19 and DZIP-1 was disrupted in *talp-3* or *ankr-26* single mutant, and the fluorescence complementation signal between ANKR-26 and DYF-19 was also disrupted in *dzip-1* single mutant. (B) BiFC signals among DZIP-1, ANKR-26 and TALP-3 in amphid cilia of worms with indicated genetic background. In *dyf-19* or *talp-3* single mutants, the fluorescence complementation signal between DZIP-1 and ANKR-26 was comparable to that in WT. In *ankr-26* or *dyf-19* single mutants, the fluorescence complementation signal between TALP-3 and DZIP-1 was comparable to that in WT. In *dzip-1* single mutant, the fluorescence complementation signal between ANKR-26 and TALP-3 was also comparable to that in WT. Scale bars: 5 µm.

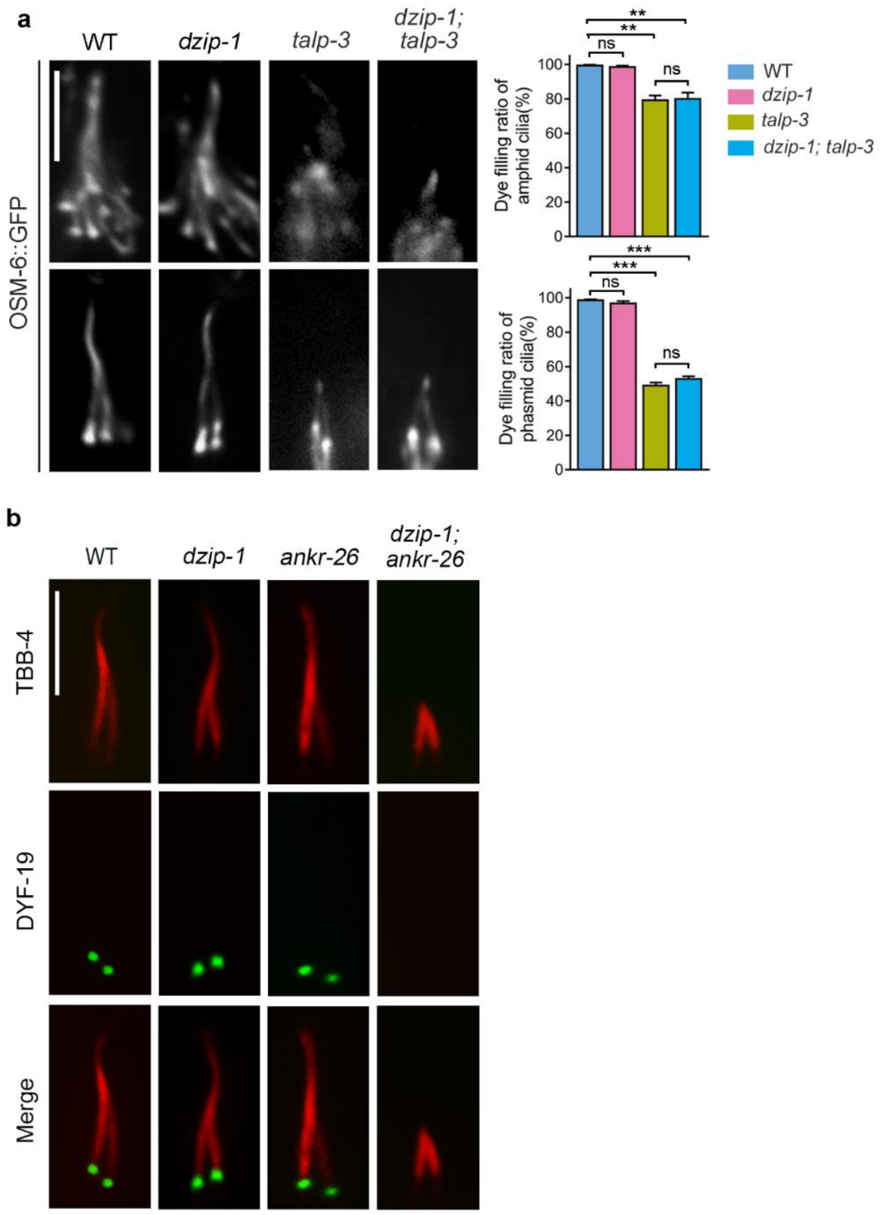

Figure S3. The function of DZIP-1 in ciliogenesis in *C. elegans*. (A) There is no genetic interaction between DZIP-1 and TALP-3 in ciliogenesis. Left panel: the morphology of cilia indicated by GFP-tagged OSM-6. Right panel: Dye-filling ratio of amphid cilia and phasmid cilia in indicated genetic background worms. The dye-filling ratio of *dzip1; talp-3* double mutants is comparable to *talp-3* single mutants. Data are presented as the mean ± SEM (n > 300 for each genotype from three independent experiments). ns, P > 0.05; **, P < 0.01; ***, P < 0.001. Scale bars = 5 µm. (B) Fluorescence micrographs of cilia labelled with the axonemal marker TBB-4::mCherry in the indicated genetic backgrounds. The cilia in *dzip-1* or *ankr-26* single mutant were comparable to those of WT, however, in *dzip-1; ankr-26* double mutants, the cilia were severely truncated.

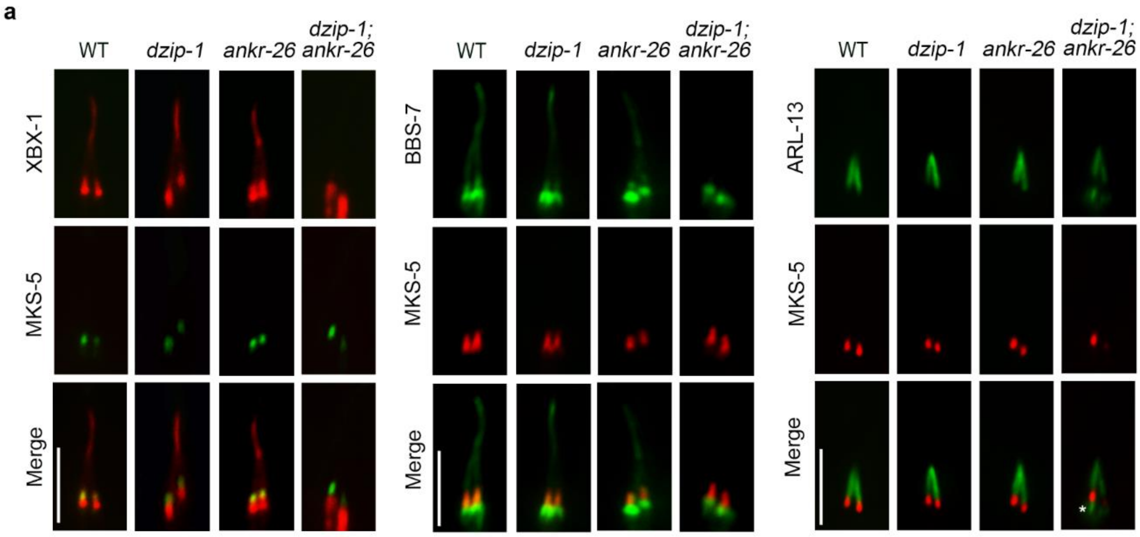

Figure S4. The ciliary entry of XBX, BBS-7 and ARL-13 is compromised in *dzip-1; ankr-26* double mutants. (A) Left and middle panel: Co-labelling with TZ markers revealed that XBX-1 and BBS-7 were able to enter ciliary compartment normally in *dizp-1* or *ankr-26* single mutants, but were restricted below the TZ in *dzip-1; ankr-26* double mutants. Right panel: The localization of ARL-13 in cilia in indicated background. Abnormal accumulation (white asterisk) of ARL-13 below the TZ was observed in *dzip-1; ankr-26* double mutants, but not in *dizp-1* or *ankr-26* single mutants. Scale bars = 5 μm.

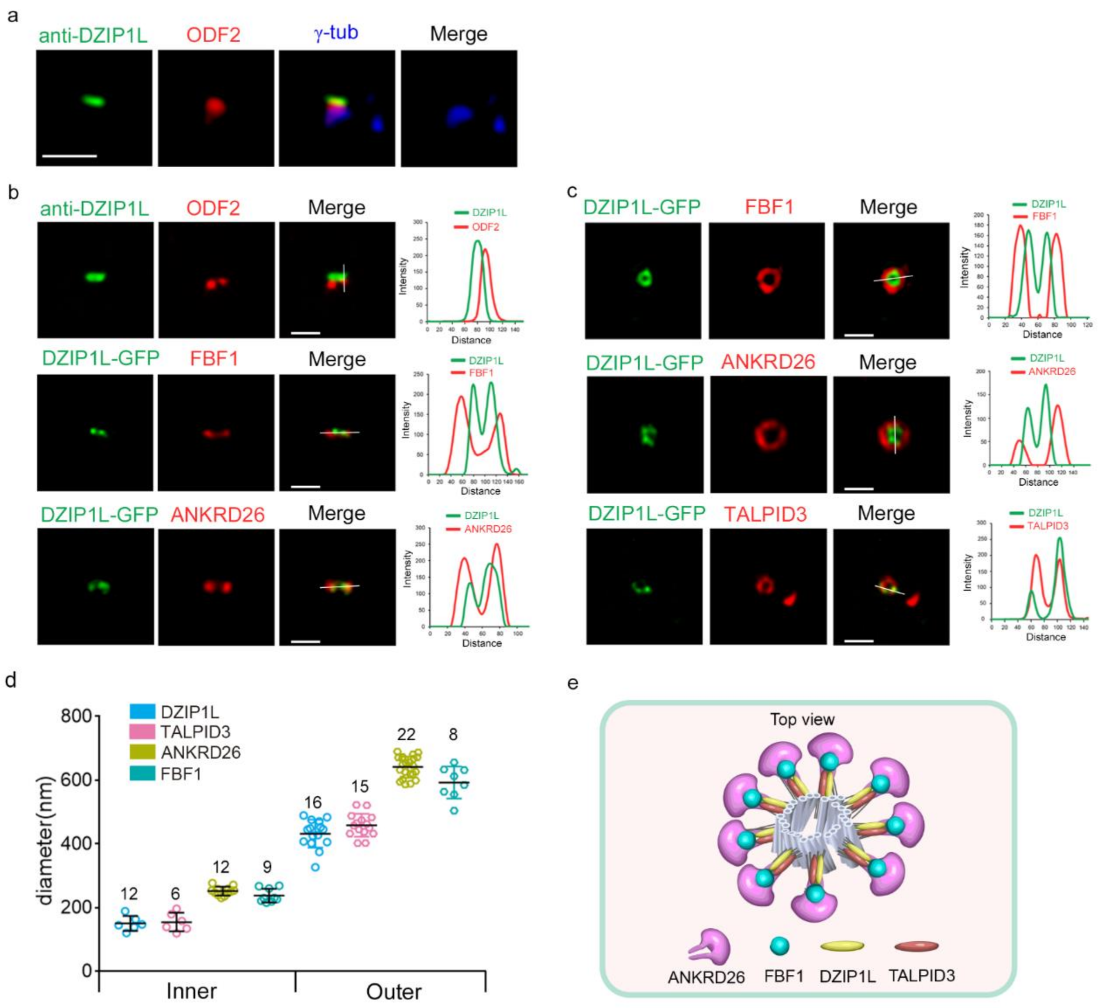

Figure S5. The localization of DZIP1L in human RPE cells. (A) DZIP1L labeled by anti-DZIP1L was localized above the sub-distal appendages labelled by ODF2. Scale bars: 1μm. (B and C) Spatial localization of DZIP1L at the distal centriole revealed by 3D-SIM (three-dimensional structured illumination microscopy). (B) DZIP1L labeled by anti-DZIP1L was localized above ODF2, but GFP antibody labeled DZIP1L-GFP was localized at the similar level as TF proteins FBF1and ANKRD26 on the Z-axis. Scale bars: 200nm. (C) GFP antibody labeled DZIP1L-GFP is organized in toroid and co-localizes with centriole distal component TALPID3. The diameter of DZIP1L-GFP toroid is smaller than TF proteins FBF1 and ANKRD26. (D) Inner and outer diameters of rings composed of DZIP1L-GFP, TALPID3, ANKRD26 and FBF1 were measured. Data are presented as the mean ± SEM (The number of cell examined are indicated above each dataset). (E) Cartoon illustrating the spatial relationship of DZIP1L, ANKRD26, TALPID3 and FBF1 in the distal centriole/basal body from the top view.

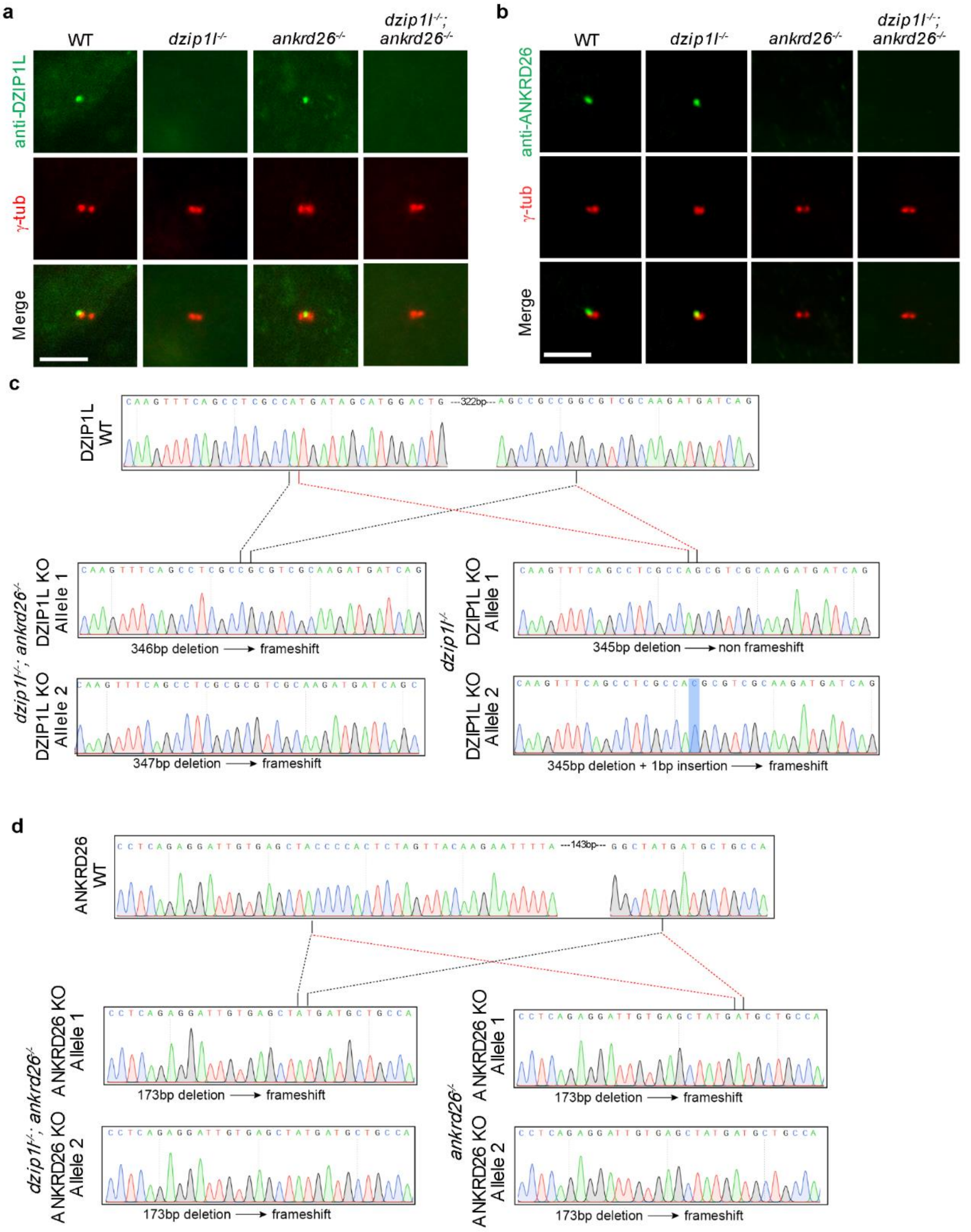

Figure S6. CRISPR-Cas9 gene editing information on the knockout of *Dzip1l* and *Ankrd26* genes in human RPE cells. (A) DZIP1L signal stained by anti-DZIP1L was lost in *dzip1l* single mutants and *dzip1l; ankrd26* double mutants. (B) ANKRD26 signal stained by anti-ANKRD26 was lost in *ankrd26* single mutants and *dzip1l; ankrd26* double mutants. (C) Gene editing information in DZIP1L was identified by sequencing. (D) Gene editing information in ANKRD26 was identified by sequencing.

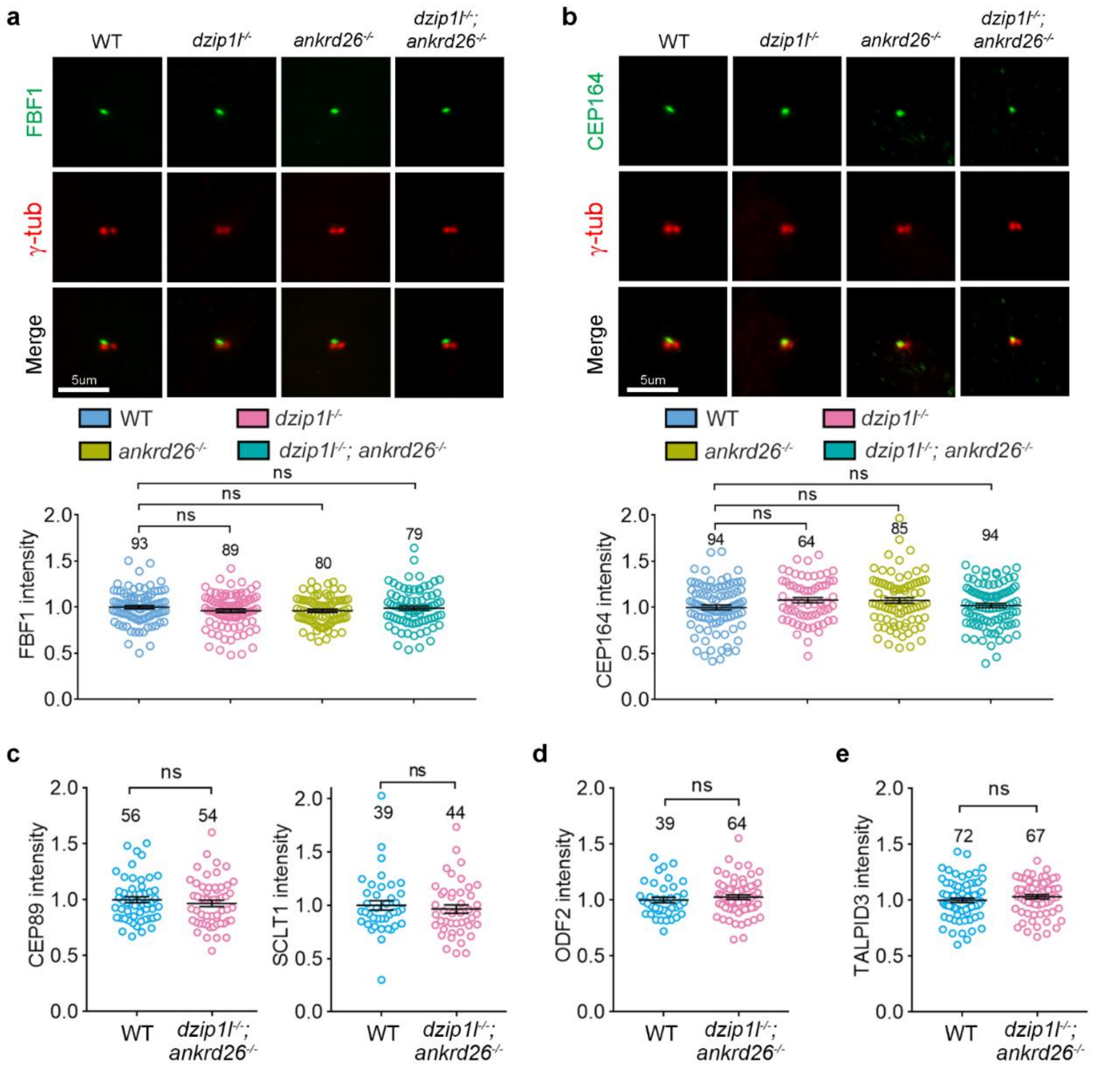

Fig. S7. Localization of core distal centriole proteins observed by confocal microscopy in human RPE-1 cells with the indicated genetic background. (A) Under confocal microscopy, the signal intensity of FBF1 in *dzip1l*$^{-/-}$; *ankrd26*$^{-/-}$ double mutant cells was comparable to that in either single mutant cells or wild-type cells. Upper panel: Representative confocal images of FBF1 signal in cells with the indicated background. Lower panel: The statistical plot of fluorescence signal intensity of FBF1 in cells of indicated genetic background. (B) Under confocal microscopy, the signal intensity of CEP164 in *dzip1l*$^{-/-}$; *ankrd26*$^{-/-}$ double mutant cells was comparable to that in either single mutant cells or wild-type cells. Upper panel: Representative confocal images of CEP164 signal in cells with the indicated background. Lower panel: The statistical plot of fluorescence signal intensity of CEP164 in cells of indicated genetic background. (C) Statistical plot of fluorescence signal intensity of distal appendage components (CEP89 and SCLT1) in cells of the indicated genetic background. (D) Statistical plot of fluorescence signal intensity of subdistal appendage components (ODF2) in cells of the indicated genetic background. (E) Statistical plot of fluorescence signal intensity of distal centriole protein (TALPID3) in cells of the indicated genetic background. Data are presented as the mean ± SEM (The numbers of cells examined is indicated above each dataset). Significant differences were identified by Mann-Whitney test. ns, $P > 0.05$. Scale bars: 5 µm.

Video S1: 3D movie of the spatial localization of DZIP1(Red) and FBF1(Green) in ciliated cells.

Video S2: 3D movie of the spatial localization of DZIP1(Red) and FBF1(Green) in non-ciliated cells.

Video S3: 3D movie of the spatial localization of DZIP1(Red) and ANKRD26(Green) in ciliated cells.

Video S4: 3D movie of the spatial localization of DZIP1(Red) and ANKRD26(Green) in non-ciliated cells.

Table S1: Worm strains used in this study.

Strain name	Genotype
QWL900	*dzip-1 (syb947) IV*
QWL901	*qwaEx300 [Parl-13 :: DZIP-1 :: GFP+MKS-5::Mcherry +pRF4]*
QWL902	*qwaEx301 [Parl-13 :: DZIP-1*$^{\Delta(66\text{-}93)}$ *:: GFP+MKS-5::Mcherry +pRF4]*
QWL903	*qwaEx302 [Parl-13 :: DZIP-1*$^{(66\text{-}93)}$ *:: GFP+MKS-5::Mcherry +pRF4]*
QWL904	*qwaEx303 [Pdzip-1 :: GFP+pRF4]*
QWL905	*qwaEx304 [Pdzip-1 :: DZIP-1 :: GFP+MKS-5::Mcherry +pRF4]*
QWL906	*qwaEx305 [Parl-13 :: DZIP-1 :: GFP +OSM-6::Mcherry+pRF4]*
QWL907	*qwaEx306 [Parl-13 :: DZIP-1 :: GFP +DYF-19::Mcherry+pRF4]*
QWL908	*qwaEx307 [Parl-13 :: DZIP-1 :: GFP +ANKR-26::Mcherry+pRF4]*
QWL909	*qwaEx308 [Parl-13 :: DZIP-1 :: GFP +MKS-5::Mcherry+pRF4]*
QWL911	*qwaEx310 [Parl-13 :: DZIP-1 :: GFP +RPI-2::Mcherry+pRF4]*
QWL912	*qwaEx311 [Parl-13 :: DZIP-1 :: GFP +TALP-3::Mcherry+pRF4]*
QWL913	*qwaEx312 [Parl-13 :: DZIP-1 :: GFP +NPHP-1::Mcherry+pRF4]*
QWL914	*qwaEx313 [ANKR-26::Venus1-173+DZIP-1::Venus155-238+MKS-5::mcherry+pRF4]*
QWL915	*qwaEx314 [DYF-19::Venus1-173+DZIP-1::Venus155-238+MKS-5::mcherry+pRF4]*
QWL916	*qwaEx315 [TALP-3::Venus1-173+DZIP-1::Venus155-238+MKS-5::mcherry+pRF4]*
SP2101	*mnIs17 [OSM6::GFP]V*
QWL904	*dzip-1 (syb947) IV; mnIs17 [osm-6::GFP] V*
QWL905	*dzip-1 (syb947) IV; qwaEx300 [Parl-13 :: DZIP-1 :: GFP+MKS-5::Mcherry +pRF4]*
VC1268	*ankr-26 (gk567)II;*
QW215	*ankr-26 (gk567)II; mnIs17 [osm-6::GFP] V*
QWL917	*dzip-1 (syb947) IV; ankr-26 (gk567)II; mnIs17 [osm-6::GFP] V*
QWL918	*ankr-26 (gk567)II; qwaEx308 [Parl-13 :: DZIP-1 :: GFP +MKS-5::Mcherry+pRF4]*
OD192	*hyls-1(tm3067)V;*
QWL919	*hyls-1(tm3067)V; qwaEx308 [Parl-13 :: DZIP-1 :: GFP +MKS-5::Mcherry+pRF4]*
VC2343	*gasr-8 (gk1232)V;*
QWL920	*gasr-8 (gk1232)V; qwaEx308 [Parl-13 :: DZIP-1 :: GFP +MKS-5::Mcherry+pRF4]*
QWL921	*dyf-19(ko104)V;*
QWL922	*dyf-19(ko104)V; qwaEx308 [Parl-13 :: DZIP-1 :: GFP +MKS-5::Mcherry+pRF4]*
QWL923	*talp-3(jhu511)IV; qwaEx308 [Parl-13 :: DZIP-1 :: GFP +MKS-5::Mcherry+pRF4]*
QWL924	*ankr-26 (gk567)II; talp-3(jhu511)IV; qwaEx308 [Parl-13 :: DZIP-1 :: GFP +MKS-5::Mcherry+pRF4]*
DAM281	*ccep-290(tm4927)I;*
QWL925	*ccep-290(tm4927)I; qwaEx308 [Parl-13 :: DZIP-1 :: GFP +MKS-5::Mcherry+pRF4]*
QWL926	*mks-5(tm3100) II; qwaEx308 [Parl-13 :: DZIP-1 :: GFP +MKS-5::Mcherry+pRF4]*
PT1408	*Ex [OSM-5::GFP+pRF4]*
QWL927	*dzip-1 (syb947) IV; Ex [OSM-5::GFP+pRF4]*

QWL928	*ankr-26 (gk567)II; Ex [OSM-5::GFP+pRF4]*
QWL929	*dzip-1 (syb947) IV; ankr-26 (gk567)II; Ex [OSM-5::GFP+pRF4]*
QWL11	*qwaEx210 [CHE-11::GFP+pRF4]*
QWL930	*dzip-1 (syb947) IV; qwaEx210 [CHE-11::GFP+pRF4]*
QWL931	*ankr-26 (gk567)II; qwaEx210 [CHE-11::GFP+pRF4]*
QWL932	*dzip-1 (syb947) IV; ankr-26 (gk567)II; qwaEx210 [CHE-11::GFP+pRF4]*
OSM-3::GFP	*Ex [OSM-3::GFP+pRF4]*
QWL933	*dzip-1 (syb947) IV; Ex [OSM-3::GFP+pRF4]*
QWL934	*ankr-26 (gk567)II; Ex [OSM-3::GFP+pRF4]*
QWL935	*dzip-1 (syb947) IV; ankr-26 (gk567)II; Ex [OSM-3::GFP+pRF4]*
QWL936	*Ex [KAP-1::GFP+pRF4]*
QWL937	*dzip-1 (syb947) IV; Ex [KAP-1::GFP+pRF4]*
QWL938	*ankr-26 (gk567)II; Ex [KAP-1::GFP+pRF4]*
QWL939	*dzip-1 (syb947) IV; ankr-26 (gk567)II; Ex [KAP-1::GFP+pRF4]*
YH652	*Ex [GFP::MKS-3+XBX-1::tdTomato+pRF4]*
QWL940	*dzip-1 (syb947) IV; Ex [GFP::MKS-3+XBX-1::tdTomato+pRF4]*
QWL941	*ankr-26 (gk567)II; Ex [GFP::MKS-3+XBX-1::tdTomato+pRF4]*
QWL942	*dzip-1 (syb947) IV; ankr-26 (gk567)II; Ex [GFP::MKS-3+XBX-1::tdTomato+pRF4]*
QWL19	*qwaEx211 [BBS-7::GFP+MKS-5::mCherry+pRF4]*
QWL943	*dzip-1 (syb947) IV; qwaEx211 [BBS-7::GFP+MKS-5::mCherry+pRF4]*
QWL944	*ankr-26 (gk567)II; qwaEx211 [BBS-7::GFP+MKS-5::mCherry+pRF4]*
QWL945	*dzip-1 (syb947) IV; ankr-26 (gk567)II; qwaEx211 [BBS-7::GFP+MKS-5::mCherry+pRF4]*
QWL405	*Ex [OSM-9::gfp+MKS-5::mCherry]*
QWL946	*dzip-1 (syb947) IV; Ex [OSM-9::gfp+MKS-5::mCherry]*
QWL947	*ankr-26 (gk567)II; Ex [OSM-9::gfp+MKS-5::mCherry]*
QWL948	*dzip-1 (syb947) IV; ankr-26 (gk567)II; Ex [OSM-9::gfp+MKS-5::mCherry]*
CX3344	*Ex [ODR-10::gfp+MKS-5::mCherry]*
QWL949	*dzip-1 (syb947) IV; Ex [ODR-10::gfp+MKS-5::mCherry]*
QWL950	*ankr-26 (gk567)II; Ex [ODR-10::gfp+MKS-5::mCherry]*
QWL951	*dzip-1 (syb947) IV; ankr-26 (gk567)II; Ex [ODR-10::gfp+MKS-5::mCherry]*
ZP1362	*Ex [DYF-19::GFP+MKS-5::mCherry+pRF4]*
QWL952	*dzip-1 (syb947) IV; Ex [DYF-19::GFP+MKS-5::mCherry+pRF4]*
QW197	*ankr-26 (gk567)II; Ex [DYF-19::GFP+MKS-5::mCherry+pRF4]*
QWL953	*dzip-1 (syb947) IV; ankr-26 (gk567)II; Ex [DYF-19::GFP+MKS-5::mCherry+pRF4]*
QW83	*Ex [Parl-13::TALP-3::GFP+Parl-13::MKS-5::mCherry+pRF4]*
QWL954	*dzip-1 (syb947) IV; Ex [Parl-13::TALP-3::GFP+Parl-13::MKS-5::mCherry+pRF4]*
QWL955	*ankr-26 (gk567)II; Ex [Parl-13::TALP-3::GFP+Parl-13::MKS-5::mCherry+pRF4]*
QWL956	*dzip-1 (syb947) IV; ankr-26 (gk567)II; Ex [Parl-13::TALP-3::GFP+Parl-13::MKS-5::mCherry+pRF4]*

QWL957	*dyf-19(ko104)V; qwaEx313 [ANKR-26::Venus1-173+DZIP-1::Venus155-238+MKS-5::mcherry+pRF4]*
QWL958	*talp-3(jhu511)IV; qwaEx313 [ANKR-26::Venus1-173+DZIP-1::Venus155-238+MKS-5::mcherry+pRF4]*
QWL959	*ankr-26(gk567)II; qwaEx314 [DYF-19::Venus1-173+DZIP-1::Venus155-238+MKS-5::mcherry+pRF4]*
QWL960	*talp-3(jhu511)IV; qwaEx314 [DYF-19::Venus1-173+DZIP-1::Venus155-238+MKS-5::mcherry+pRF4]*
QWL961	*ankr-26(gk567)II; talp-3(jhu511)IV; qwaEx314 [DYF-19::Venus1-173+DZIP-1::Venus155-238+MKS-5::mcherry+pRF4]*
QWL962	*dyf-19(ko104)V; qwaEx315 [TALP-3::Venus1-173+DZIP-1::Venus155-238+MKS-5::mcherry+pRF4]*
QWL963	*ankr-26(gk567)II; qwaEx315 [TALP-3::Venus1-173+DZIP-1::Venus155-238+MKS-5::mcherry+pRF4]*
QW295	*Ex [ANKR-26::Venus1-173+TALP-3::Venus155-238+MKS-5::mcherry+pRF4]*
QWL964	*dzip-1 (syb947) IV; Ex [ANKR-26::Venus1-173+TALP-3::Venus155-238+MKS-5::mcherry+pRF4]*
QW311	*Ex [ANKR-26::Venus1-173+DYF-19::Venus155-238+MKS-5::mcherry+pRF4]*
QWL965	*dzip-1 (syb947) IV; Ex [ANKR-26::Venus1-173+DYF-19::Venus155-238+MKS-5::mcherry+pRF4]*
QW296	*Ex [TALP-3::Venus1-173+DYF-19::Venus155-238+MKS-5::mcherry+pRF4]*
QWL966	*dzip-1 (syb947) IV; Ex [TALP-3::Venus1-173+DYF-19::Venus155-238+MKS-5::mcherry+pRF4]*
QWL967	*ankr-26(gk567)II; Ex [TALP-3::Venus1-173+DYF-19::Venus155-238+MKS-5::mcherry+pRF4]*
QWL968	*dzip-1 (syb947) IV; ankr-26(gk567)II; Ex [TALP-3::Venus1-173+DYF-19::Venus155-238+MKS-5::mcherry+pRF4]*
QWL969	*qwaEx316 [DYF-19::GFP+TBB-4::mCherry]*
QWL970	*dzip-1(syb947) IV; qwaEx316 [DYF-19::GFP+TBB-4::mCherry]*
QWL971	*ankr-26 (gk567) II; qwaEx316 [DYF-19::GFP+TBB-4::mCherry]*
QWL972	*dzip-1(syb947) IV; ankr-26 (gk567) II; qwaEx316 [DYF-19::GFP+TBB-4::mCherry]*
QWL973	*qwaEx317 [Parl-13::hDZIP1(1-358aa)::GFP+Parl-13:: MKS-5::mCherry+pRF4]*
QWL974	*qwaEx318 [Parl-13::hDZIP1L (1-415aa)::GFP+Parl-13:: MKS-5::mCherry+pRF4]*
QWL975	*qwaEx319 [Parl-13::hDZIP-1(AQ2GH)::GFP+Parl-13:: MKS-5::mCherry+pRF4]*
QWL976	*dzip-1(syb947) IV; ankr-26 (gk567) II; qwaEx319 [Parl-13::hDZIP-1(AQ2GH)::GFP+Parl-13:: MKS-5::mCherry+pRF4]*
QWL977	*qwaEx320 [Parl-13::ARL-13::GFP+Parl-13:: MKS-5::mCherry+pRF4]*
QWL978	*dzip-1(syb947) IV; qwaEx320 [Parl-13::ARL-13::GFP+Parl-13:: MKS-5::mCherry+pRF4]*
QWL979	*ankr-26 (gk567) II; qwaEx320 [Parl-13::ARL-13::GFP+Parl-13:: MKS-5::mCherry+pRF4]*
QWL980	*dzip-1(syb947) IV; ankr-26 (gk567) II; qwaEx320 [Parl-13::ARL-13::GFP+Parl-13:: MKS-5::mCherry+pRF4]*
QWL990	*Is [PKD-2::GFP; CC::GFP];EX[MKS-5::mCherry+pRF4]*
QWL991	*dzip-1 (syb947) IV; Is [PKD-2::GFP; CC::GFP];EX[MKS-5::mCherry+pRF4]*
QWL992	*ankr-26 (gk567)II; Is [PKD-2::GFP; CC::GFP];EX[MKS-5::mCherry+pRF4]*
QWL993	*dzip-1 (syb947) IV; ankr-26 (gk567)II; Is [PKD-2::GFP; CC::GFP];EX[MKS-5::mCherry+pRF4]*
